# Platelet abnormalities in autoimmune thyroid diseases: A systematic review and meta-analysis

**DOI:** 10.3389/fimmu.2022.1089469

**Published:** 2022-12-22

**Authors:** Yu-tian Cao, Kai-yu Zhang, Jing Sun, Yan Lou, Tian-su Lv, Xinyi Yang, Wen-hui Zhang, Jiang-yi Yu, Qi-biao Wu, Xi-qiao Zhou

**Affiliations:** ^1^ Department of Endocrinology, Jiangsu Province Hospital of Chinese Medicine, Affiliated Hospital of Nanjing University of Chinese Medicine, Nanjing, China; ^2^ The First Clinical Medical College of Nanjing University of Chinese Medicine, Nanjing, China; ^3^ State Key Laboratory of Quality Research in Chinese Medicines and Faculty of Chinese Medicine, Macau University of Science and Technology, Taipa, Macao, Macao SAR, China

**Keywords:** autoimmune thyroid disease, Grave’s disease, Hashimoto autoimmune thyroiditis, platelet count, mean platelet volume, systematic review, meta - analysis

## Abstract

**Background:**

Some degree of platelet index abnormality has been found clinically in the autoimmune thyroid disease (AITD), but the findings are not uniform.

**Methods:**

The PubMed, Web of Science, Cochrane Library, and Embase databases were searched for relevant articles published up to August 16th, 2022, with no restrictions on the language of the articles. Reference lists of eligible articles were also searched. A random effect model was used to pool the standardized mean difference (SMD) and 95% confidence interval (95% CI) of platelet count (PLT), mean platelet volume (MPV), and platelet distribution width (PDW) between AITD patients and healthy controls, and subgroup analyses were performed.

**Results:**

A total of 19 articles with 6173 people (3824 AITD patients and 2349 healthy people) were included in the meta-analysis. The results showed that PLT and MPV values were significantly increased in AITD patients when compared with healthy people (SMD: 0.164, 95% CI: 0.044 to 0.285; SMD: 0.256, 95% CI: 0.013 to 0.500), while no significant difference was found in PDW between the AITD group and the control group (SMD: 0.060, 95% CI: -0.164 to 0.284). Subgroup analysis according to disease type and thyroid function revealed that for PLT, this difference was only found in the Hashimoto’s thyroiditis (HT) and hypothyroid groups, but not in the Graves’ disease (GD) and hyperthyroid groups. For MPV, the results were the opposite of those for PLT: MPV was significantly higher in the GD, hyperthyroid, and euthyroid groups than in the control group, but not in the HT and hypothyroid groups. Sensitivity analysis showed that the stability of the pooled MPV was not good. No publication bias was found.

**Conclusions:**

PLT and MPV are significantly elevated in patients with AITD, with PLT being more significantly elevated in HT and hypothyroidism, and MPV being more significantly increased in GD and hyperthyroidism. Appropriate clinical attention can be paid to the thyroid function of patients when abnormal platelet indices are found, and conversely, the consequences of abnormal platelet parameters such as elevated MPV lead to an increased occurrence of cardiovascular events, which should also be addressed in the AITD population.

**Systematic review registration:**

https://www.crd.york.ac.uk/PROSPERO/, identifier CRD42022341823.

## Introduction

Autoimmune thyroid disease (AITD) is the most common autoimmune disease, mainly including Graves’ disease (GD) and Hashimoto’s thyroiditis (HT). GD is the most common cause of hyperthyroidism, which is mainly caused by thyrotropin receptor autoantibodies overstimulating thyroid-stimulating hormone receptors. Its clinical manifestations are mainly goiter and thyrotoxicosis, and some patients also have ocular abnormalities and localized dermopathy (1). HT is a disease characterized by thyroid specific autoantibodies (mainly including thyroid peroxidase antibody and thyroglobulin antibody), which mainly manifest as primary hypothyroidism caused by thyroid damage (2). The specific pathogenesis of AITD is not fully understood, but the interaction between genetic and environmental factors is key to the development of the disease, leading to dysfunction of lymphocytes and antigen-presenting cells, which disrupts immune homeostasis and causes thyroid autoimmunity (1, 2).

As a multifunctional cell, platelets have an important role in the regulation of immunity and inflammation, in addition to their hemostatic function (3). Platelets may be involved in the pathogenesis of AITD, and patients with AITD also have some degree of abnormal platelet parameters. Recent studies have found a higher platelet activation level in AITD (4); GD is associated with higher levels of activated lymphocytes and platelet-lymphocyte aggregates (5). In addition, it is not uncommon for AITD patients to be clinically complicated by immune thrombocytopenia (6), while reactive thrombocytosis has also been found in the HT population (7). Mean platelet volume (MPV), one of the indicators of platelet activation, meta-analysis has shown that there is a correlation between the increase MPV and the risk of thrombosis and cardiovascular events (8). Some studies found that MPV in AITD patients increased (9, 10), which increased the incidence and mortality of cardiovascular events, while others have concluded that MPV in the ATID group was not significantly different from that in the control group (11).

In view of these inconsistent findings, we conducted a meta-analysis to provide more comprehensive conclusions on changes in platelet parameters in AITD. As a simple and inexpensive laboratory indicator, studying the correlation between platelet parameters and AITD may provide broader clinical ideas for the prevention, diagnosis, and treatment of AITD and related platelet disorders.

## Methods

### Search strategy and selection criteria

This meta-analysis was conducted following the Meta-analysis of Observational Studies in Epidemiology (MOOSE) reporting guideline (12) and the Preferred Reporting Items for a Systematic Review and Meta-analysis (PRISMA) guideline (**Tables S1, S2**) (13). The protocol for this meta-analysis was registered with PROSPERO (CRD42022341823).

We searched the PubMed, Web of Science, Cochrane Library, and Embase databases for relevant articles published up to August 16th, 2022. Our search strategy consisted of MeSH terms and entry terms with no restrictions on the language of the articles. We also scanned the reference lists of eligible articles for additional eligible articles that were not retrieved during the literature search. The details of the search strategy are presented in **Table S3**.

The inclusion criteria were as follows (1): Patients: adult AITD patients (2); Control: adult in good health (3); Outcomes: platelet count (PLT), MPV, and platelet distribution width (PDW) (4); Study type: case control studies, cohort studies, and cross sectional studies (5); Studies included at least 20 patients to obtain good reliability.

The exclusion criteria were as follows (1): Animal experiments, review, conference abstracts, case reports and meta-analyses (2); Combined with severe primary systemic diseases such as cancer, acquired immune deficiency syndrome (3); Pregnant or lactating women, hematological diseases, and any other diseases could interfere with the platelet parameters (4); Subjects who had undergone thyroid surgery or iodine-131 therapy (5); Full-text or sufficient data could not be extracted.

### Data collection and extraction

Studies from the database were managed using EndNote X9 software to remove duplicate articles. The included articles based on inclusion and exclusion criteria were screened by T-SL and X-YY, who worked independently. The disagreement was discussed with another author (K-YZ) and subsequently resolved *via* consensus. Extracted research information includes (1): Background information: first author, publication year, country, study design, sample size, age, sex (2); Platelet parameters: PLT, MPV, and PDW. Values were presented as mean ± standard deviation (SD). When relevant data were missing, the corresponding author was contacted to obtain the information. If the same study contained multiple groups of available data, it would be divided into multiple independent individuals for data extraction. The process was performed independently by T-SL and X-YY. Any disagreements were resolved by discussion and consensus.

### Quality assessment

According to the recommendations of the Agency for Healthcare Research and Quality (AHRQ) for the quality evaluation criteria of observational studies, the Newcastle-Ottawa Scale (NOS) was used to evaluate cohort studies and case control studies (14–16), and the AHRQ methodology checklist was used to evaluate cross sectional studies (17). The NOS evaluates the quality of research from three aspects: the selection of the study groups; the comparability of the groups; and the ascertainment of either the exposure or outcome of interest for case control or cohort studies, respectively (**Methods 1, 2**). The quality of the study was assessed as follows: low quality = 0-3 stars; moderate quality = 4-6 stars; and high quality = 7-9 stars (18). The AHRQ methodology checklist includes 11 items: the definition of information source; inclusion and exclusion criteria; time period and continuity for identifying patients; blinding of personnel; assessments for quality assurance; confounding and missing data; and response rates and completeness of patients (**Methods 3**). If the answer is “unclear” or “no”, the item score is “0”. If the answer is “yes”, the score of the item is “1”. The quality of the study was assessed as follows: low quality = 0-3; moderate quality = 4-7; and high quality = 8-11 (17, 19). The quality of eligible articles was independently assessed by two investigators (T-SL and X-YY). Any disagreement was resolved by the third investigator (K-YZ).

### Data synthesis and data analysis

The standardized mean difference (SMD) and 95% confidence interval (95% CI) of PLT, MPV, and PDW between AITD patients and healthy controls in each study were calculated and estimated. For studies using median, range and/or interquartile range, we used the methods proposed by Wan et al. (20) and Luo et al. (21) to estimate the sample mean and SD. Positive SMDs indicated higher platelet parameter values in AITD patients. If the changes in the outcomes were not reported. We used the conversion formulas recommended in the Cochrane Handbook Version 6.4 to calculate the changes in outcomes (22).

The I^2^ statistic was used to quantitatively evaluate the heterogeneity of studies. Studies with I² of 0-25%, 25-75%, and > 75% were considered to have low, moderate, and high heterogeneity, respectively. A random effect model was applied regardless of heterogeneity. Meta-regression analyses were performed to determine the source of heterogeneity. According to the disease type, thyroid function, quality assessment, region, and study type, we conducted subgroup analyses. The groups were as follows: GD, HT, and AITD; Hyperthyroid/Before treatment, Hypothyroid/Before treatment, Euthyroid/After treatment, and Unknown; High quality (NOS = 7-9 stars or AHRQ score = 8-11), Moderate quality (NOS = 4–6 stars or AHRQ score = 4-7), and Low quality (NOS = 0-3 stars or AHRQ score = 0-3); Turkey, China, and Others; Case control study and Cross sectional study.

Besides, sensitivity analysis was performed to evaluate the robustness of the results by removing one single study in each turn. If 10 or more studies were included, we would use the funnel plots and Egger’s test to investigate publication bias. If there was publication bias, the trim and fill method was conducted to rectify the funnel plot asymmetry.

All statistical analyses were done with Stata Version 15.0 (StataCorp) from September 6 to 10, 2022. The results were considered significant when a 2-tailed P < 0.05.

## Results

### Search and selection result

**Figure 1:**
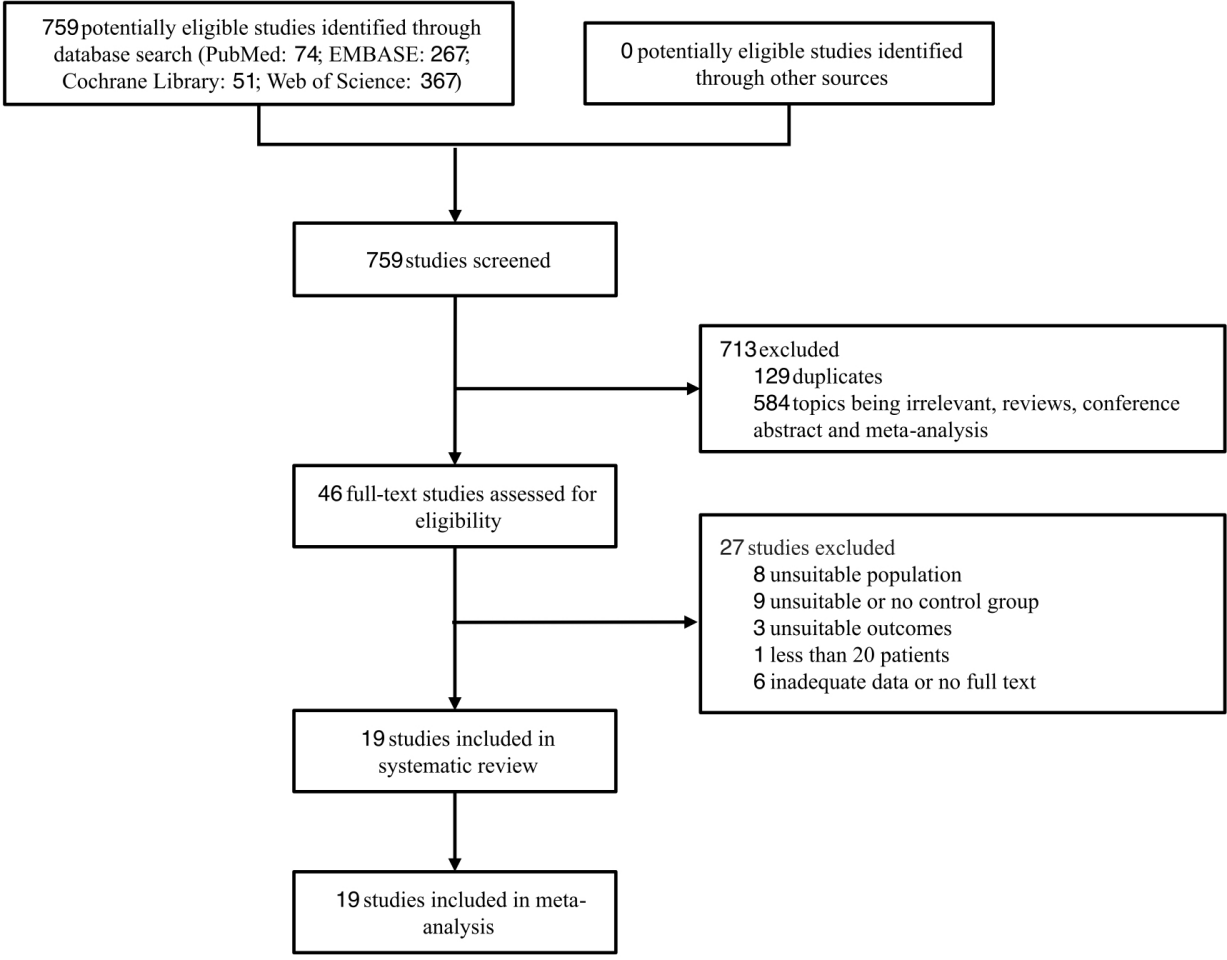
Study selection.

Through database searches, 759 records were found. After screening, 19 articles (5, 9, 11, 23–38) were finally selected for the meta-analysis. The specific screening process is shown in **Figure 1**, articles excluded after full text review are listed in Methods 4.

### Studies characteristics and quality assessment

A total of 19 articles were included, 2 of which were from the same cross sectional study (26, 27). The characteristics of the 19 included articles are shown in **Table S4**. Among them, part of the data from 4 articles (23, 30, 36, 37) were presented as median (min-max) or median (25–75th percentile), which were transformed according to the methods mentioned in Data Synthesis and Data Analysis. A comparison table of the original data and the corresponding converted data is shown in **Table S5**. Based on the NOS and AHRQ methodology checklist, 17 articles (5, 11, 23–36, 38) were classified as high quality, and 2 articles (9, 37) as moderate quality. Detailed assessment scores are shown in **Table S4**.

### Meta-analyses

**Figure 2:**
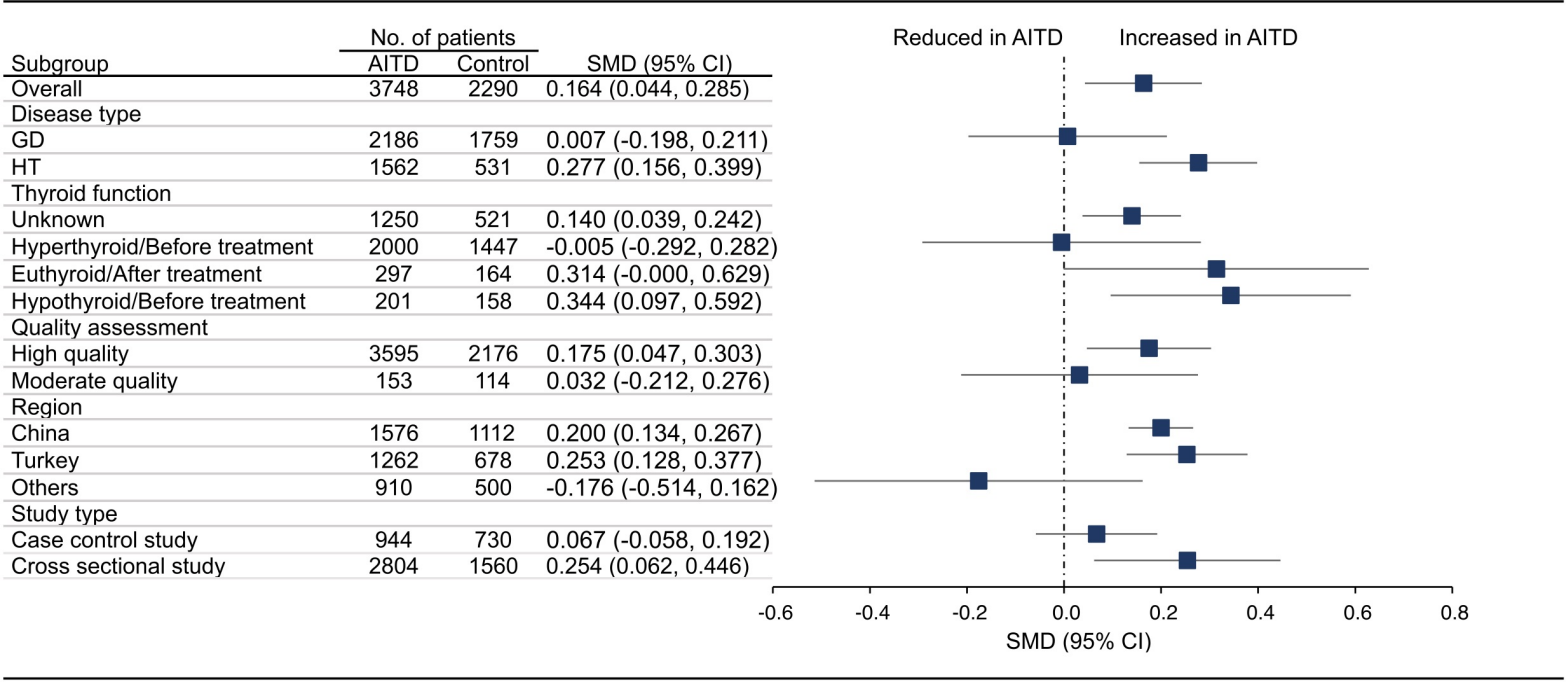
Standardized Mean Difference in PLT between the AITD Group and the Control Group. GD, Graves’ disease; HT, Hashimoto’s thyroiditis; AITD, autoimmune thyroid disease; PLT, platelet count; SMD, standardized mean difference; 95%CI, 95% confidence interval.

**Figure 3:**
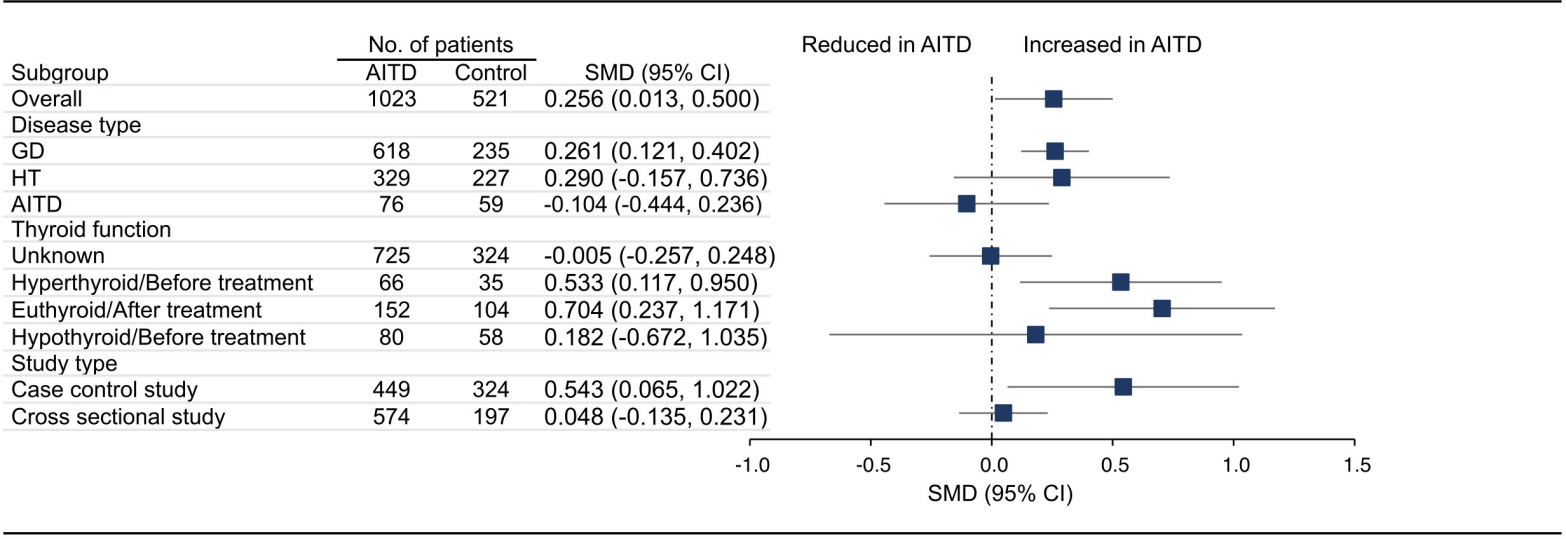
Standardized Mean Difference in MPV between the AITD Group and the Control Group. GD, Graves’ disease; HT, Hashimoto’s thyroiditis; AITD, autoimmune thyroid disease; MPV, mean platelet volume; SMD, standardized mean difference; 95%CI, 95% confidence interval.

**Figure 4:**
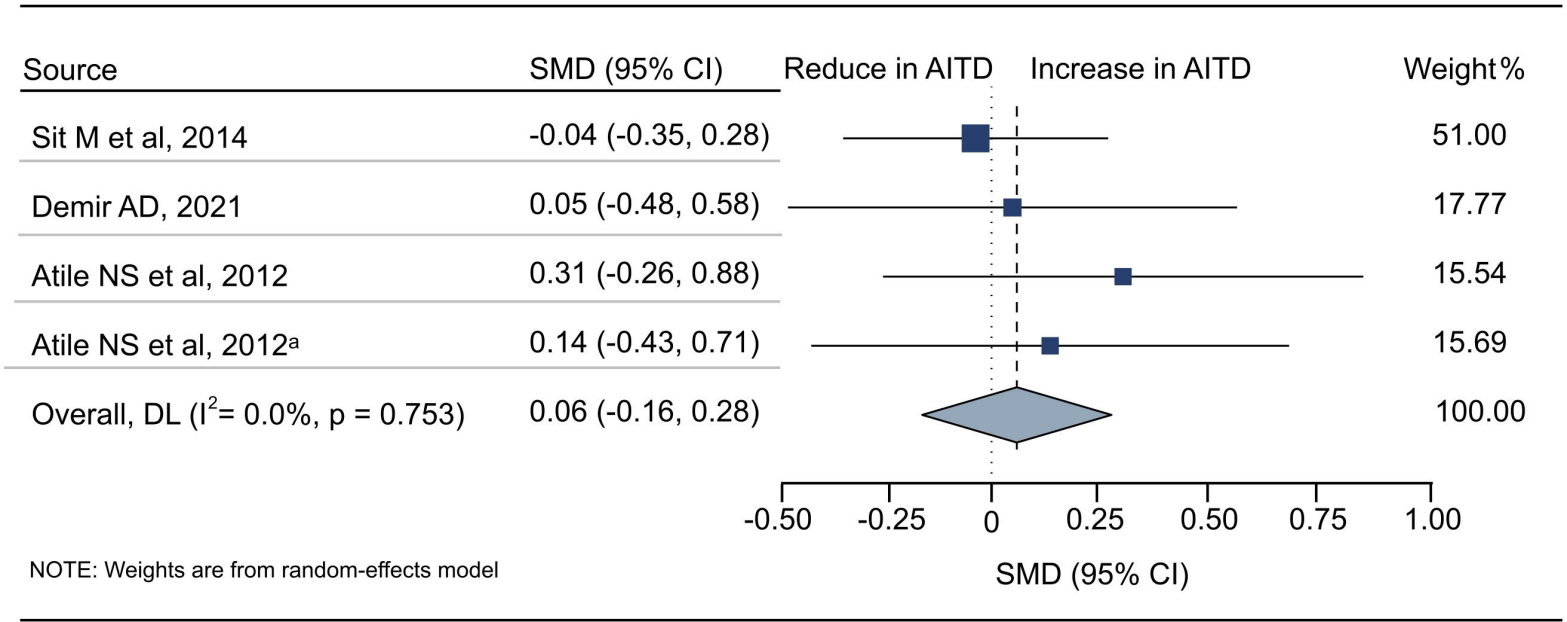
Standardized Mean Difference in PDW between the AITD Group and the Control Group. AITD, autoimmune thyroid disease; PDW, platelet distribution width; SMD, standardized mean difference; 95%CI, 95% confidence interval. a: the second available data from the same study.

18 articles (26 available data) reported the difference in PLT between the AITD group and the control group. The results showed that PLT values were significantly increased in AITD patients when compared with healthy people (SMD: 0.164, 95% CI: 0.044 to 0.285, p = 0.008, **Figure 2**). For MPV, we pooled the effects of 9 articles (14 available data) and found that MPV values were significantly increased in AITD patients when compared with healthy people (SMD: 0.256, 95% CI: 0.013 to 0.500, p = 0.039, **Figure 3**). In the analysis of PDW (3 articles, 4 available data), no significant difference was found between the AITD group and the control group (SMD: 0.060, 95% CI, -0.164 to 0.284, p = 0.601, **Figure 4**).

### Meta-regression analyses and planned subgroup analyses

Meta-regression analyses were performed according to covariates including disease type, thyroid function, quality assessment, study type, and region. The results showed that these covariates were not possible sources of heterogeneity (**Table S6**). For PDW, since the number of included studies was less than 10, no meta-regression analysis was conducted.

Furthermore, we conducted subgroup analyses according to the disease type, thyroid function, quality assessment, region, and study type. As shown in **Figure 2**, HT group had significantly higher PLT than healthy people (SMD: 0.277, 95% CI: 0.156 to 0.399), while the difference in GD group was not statistically significant (SMD: 0.007, 95% CI: -0.198 to 0.211). For thyroid function, PLT in thyroid function unknown group (SMD: 0.140, 95% CI: 0.039 to 0.242) and hypothyroid/before treatment group (SMD: 0.344, 95% CI: 0.097 to 0.592) was significantly higher than that of the control group, while there was no significant difference in hyperthyroid/before treatment group (SMD: -0.005, 95% CI: -0.292 to 0.282) and euthyroid/after treatment group (SMD: 0.314, 95% CI: -0.000 to 0.629). For studies of different quality scores, regions, and study types, the PLT in high quality group (SMD: 0.175, 95% CI: 0.047 to 0.303), China (SMD: 0.200, 95% CI: 0.134 to 0.267) and Turkey group (SMD: 0.253, 95% CI: 0.128 to 0.377), and cross sectional group (SMD: 0.254, 95% CI: 0.062 to 0.446) was significantly higher than that in the control group, while the difference in moderate quality group (SMD: 0.032, 95% CI: -0.212 to 0.276), other countries group (SMD: -0.176, 95% CI: -0.514 to 0.162), and case control group (SMD: 0.067, 95% CI: -0.058 to 0.192) was not significant (**Figures S1A–E**).

For MPV, we conducted subgroup analyses according to disease type, thyroid function, and study type. As shown in **Figure 3**, MPV in GD group (SMD: 0.261, 95% CI: 0.121 to 0.402), hyperthyroid/before treatment group (SMD: 0.533, 95% CI: 0.117 to 0.950), euthyroid/after treatment group (SMD: 0.704, 95% CI: 0.237 to 1.171), and case control group (SMD: 0.543, 95% CI: 0.065 to 1.022) was significantly higher than that of the control group, while there was no significant difference in HT group (SMD: 0.290, 95% CI: -0.157 to 0.736), AITD group (SMD: -0.104, 95% CI: -0.444 to 0.236), thyroid function unknown group (SMD: -0.005, 95% CI: -0.257 to 0.248), hypothyroid/before treatment group (SMD: 0.182, 95% CI: -0.672 to 1.035) and cross sectional group (SMD: 0.048, 95% CI: -0.135 to 0.231, **Figures S2A–E**). Due to insufficient data, we did not conduct subgroup analysis on MPV based on quality assessment and region. There were too few studies on PDW, and no subgroup analysis was conducted.

### Sensitivity analysis and publication bias

Sensitivity analysis showed that no single study significantly influenced the difference in PLT between AITD patients and healthy people. However, the stability of the pooled MPV was not good. After more than half of the studies were eliminated separately, the pooled results of the remaining studies were not statistically significant (95% CI exceeded 0, **Table S7**). In addition, funnel plots were roughly symmetrical (**Figure S3**), and Egger’s test further showed that there was no publication bias among included studies in PLT and MPV (**Table S8**, **Figure S4**). For PDW, we did not conduct publication bias analysis because fewer than 10 studies were included.

## Discussion

In this meta-analysis, we found that PLT and MPV were significantly higher in the AITD population. After further grouping according to disease type and thyroid function, PLT in the HT group, thyroid function unknown group, and hypothyroid/before treatment group was significantly higher than that of the control group; MPV in the GD group, euthyroid/after treatment group, and hyperthyroid/before treatment group was significantly higher than that in the control group.

Regarding changes in PLT in AITD, this difference was found only in the HT and hypothyroid groups, but not in the GD and hyperthyroid groups. Such a result does not seem to be a coincidence, as HT patients tend to develop hypothyroidism later in life while GD is the main cause of hyperthyroidism. Beyan et al. first reported the case of reactive thrombocytosis caused by HT. A 31-year-old man with HT stopped taking levothyroxine on his own and found that PLT increased to 715×10^9^/L during routine follow-up, and the examination showed that the patient was in a subclinical hypothyroidism state. When he started thyroid hormone replacement therapy again, PLT gradually decreased to normal (7). In addition to this case report, Bilge et al. found a significantly higher PLT in the HT group than the control group (31). Besides, several other studies, although lacking statistical differences, also suggested a slightly higher PLT in the HT group compared to controls (24, 26). HT is the major cause of hypothyroidism, and studies have found that thyroid hormone levels are also associated with PLT. One trial found an increase in PLT in hypothyroid patients two weeks after stopping thyroid hormone supplementation (39); another study showed a significant negative correlation between PLT and thyroid hormone in hypothyroid patients (10).

As mentioned in the introduction, platelets are also important players in immunity and inflammation. Activated platelets synthesize and release pro-thrombotic and pro-inflammatory factors from the granule system. Many platelet-derived factors contribute to the formation of inflammatory response, the most important of which is P-selectin, which binds to the P-selectin glycoprotein ligand-1 on endothelial or immune cells, thereby enabling platelets to bind to the inflamed endothelium, recruit circulating leukocytes, promote platelet leukocyte aggregation, and initiate an inflammatory response at the injured site (3, 40). As HT is an autoimmune thyroiditis and is characterized pathologically by an intra-thyroidal lymphocytic infiltrate, platelets are likely to be involved in its pathogenesis and cause changes in platelet parameters. Thrombopoietin produced by the liver plays an important role in megakaryocyte proliferation, differentiation, and platelet formation (41). It was found that inflammatory factors can stimulate the production of thrombopoietin, which also means that PLT may increase significantly in inflammatory states (42). In addition, elevated levels of tumor necrosis factor-α were found to lead to platelet hyperreactivity in the model of aging mouse (43). On the one hand, it may induce transcriptional reprogramming of megakaryocytes, leading to an increase in PLT (43). On the other hand, it stimulates platelet activation and adhesion, forming more platelet-leukocyte aggregates that further promote the progression of immune inflammation, forming a vicious circle. Although a study has shown a significantly higher PLT in GD (29), our results did not find a significant difference in PLT between the GD or hyperthyroid population and the healthy population. Such results are curious, as GD is also an immune disease, so why does it not show elevated PLT like HT? We speculate that it may be related to the following factors: Elevated thyroid hormone levels in GD patients enhance the phagocytosis of the reticuloendothelial system and shorten the platelet lifespan (44), which is superimposed on the increase in platelets due to immunoinflammatory factors, resulting in insignificant changes.

For MPV changes in AITD, the results were the opposite of those in PLT. The MPV was significantly higher in the GD, hyperthyroid/before treatment, and euthyroid/after treatment groups than in the control group, while it was not statistically significant in the HT and hypothyroid groups. One explanation is that the active reticuloendothelial phagocyte system in patients with GD leads to a decrease in platelets (44), while the newborn platelets are larger in size (45). However, it is noteworthy that MPV was also statistically significant in the euthyroid group, which contradicts the conventional perception that abnormal thyroid hormone levels lead to elevated MPV. MPV was found to be significantly higher in euthyroid patients with AITD than in controls (9, 10), and MPV was positively correlated with thyroid peroxidase antibody (46). These studies suggest that the mechanism of MPV abnormalities in AITD patients may be immune and inflammatory rather than hormonal disorders. Of course, this also needs to be verified in more clinical studies. The meta-analysis showed that increased MPV was strongly associated with cardiovascular events (8). MPV as one of the indicators of platelet activation, AITD patients have significantly higher levels of platelet activation (4), and thyroid dysfunction is strongly associated with thrombosis, increasing the morbidity and mortality of cardiovascular events (47–49). Therefore, appropriate clinical attention can be paid to changes in MPV in AITD patients to assess their risk factors for cardiovascular events and intervene in a timely manner. Notably, the multifaceted nature of platelets gives them a place in human health and in a considerable number of diseases, and abnormal platelet indices are not limited to AITD. In recent years, there have been an increasing number of studies related to this issue, and to our knowledge, this is the first meta-analysis to assess differences in platelet parameters between AITD and healthy populations. We have drawn some conclusions from the data analysis and hope that this will also make a small contribution to research in the field of platelets.

Even so, there were some limitations to our work. First, most of the studies were conducted in China and Turkey, and there were differences in the platelet analyzers used, which can lead to bias and made it difficult to generalize these findings to a broader scope. Second, partial summary results had great heterogeneity. Although subgroup analysis explained some of the sources of heterogeneity, there were still some that they couldn’t explain. Third, sensitivity analysis showed that the stability of the pooled MPV was not good, which limits the interpretability of our result. Finally, although we considered the effect of disease type, thyroid function, quality assessment, region, and study type on platelets, we were unable to extract sufficient data for other confounding factors, such as gender and age, for further analysis.

In conclusion, this meta-analysis showed that PLT and MPV were significantly elevated in AITD patients when compared with healthy people. Subgroup analysis based on disease type and thyroid function revealed that this elevation was more significant for PLT in the HT and hypothyroid populations and for MPV in the GD and hyperthyroid populations. The clinical findings of platelet abnormalities can appropriately focus on the patient’s thyroid function, and conversely, the consequences of platelet abnormalities should be prevented in the AITD population. However, these findings must be considered in the context of above limitations, and more clinical studies are needed to validate them.

## Data availability statement

The original contributions presented in the study are included in the article/**Supplementary Material**. Further inquiries can be directed to the corresponding author.

## Author contributions

YC and XZ conceived and designed the study. KZ, TL and XY contributed to data collection. YL, TL, WZ and JS conducted the data analysis and interpretation. YC and KZ drafted the initial manuscript. XZ, JY and QW revised the manuscript. All authors contributed to the article and approved the submitted version.
