# Corrigendum: Platelet abnormalities in autoimmune thyroid diseases: A systematic review and meta-analysis

**DOI:** 10.3389/fimmu.2023.1166711

**Published:** 2023-02-21

**Authors:** Yu-tian Cao, Kai-yu Zhang, Jing Sun, Yan Lou, Tian-su Lv, Xinyi Yang, Wen-hui Zhang, Jiang-yi Yu, Qi-biao Wu, Xi-qiao Zhou

**Affiliations:** ^1^ Department of Endocrinology, Jiangsu Province Hospital of Chinese Medicine, Affiliated Hospital of Nanjing University of Chinese Medicine, Nanjing, China; ^2^ The First Clinical Medical College of Nanjing University of Chinese Medicine, Nanjing, China; ^3^ State Key Laboratory of Quality Research in Chinese Medicines and Faculty of Chinese Medicine, Macau University of Science and Technology, Taipa, Macao, Macao SAR, China

**Keywords:** autoimmune thyroid disease, Grave’s disease, Hashimoto autoimmune thyroiditis, platelet count, mean platelet volume, systematic review, meta – analysis

In the published article, there was an error in affiliations 1 and 2. Instead of “Yu-tian Cao^1,2^, Kai-yu Zhang^1^, Jing Sun^1,2^, Yan Lou^2^, Tian-su Lv^1^, Xinyi Yang^1,2^, Wen-hui Zhang^1,2^, Jiang-yi Yu^1^, Qi-biao Wu^3^ and Xi-qiao Zhou^2^*

1. The First Clinical Medical College of Nanjing University of Chinese Medicine, Nanjing, China

2. Department of Endocrinology, Affiliated Hospital of Nanjing University of Chinese Medicine, Jiangsu Province Hospital of Chinese Medicine, Nanjing, China

3. State Key Laboratory of Quality Research in Chinese Medicines and Faculty of Chinese Medicine, Macau University of Science and Technology, Taipa, Macao, Macao SAR, China”, it should be “Yu-tian Cao^1,2^, Kai-yu Zhang^2^, Jing Sun^1,2^, Yan Lou^1^, Tian-su Lv^2^, Xinyi Yang^1,2^, Wen-hui Zhang^1,2^, Jiang-yi Yu^1^, Qi-biao Wu^3^ and Xi-qiao Zhou^1^*

1. Department of Endocrinology, Jiangsu Province Hospital of Chinese Medicine, Affiliated Hospital of Nanjing University of Chinese Medicine, Nanjing, China

2. The First Clinical Medical College of Nanjing University of Chinese Medicine, Nanjing, China

3. State Key Laboratory of Quality Research in Chinese Medicines and Faculty of Chinese Medicine, Macau University of Science and Technology, Taipa, Macao, Macao SAR, China”.

The authors apologize for this error and state that this does not change the scientific conclusions of the article in any way. The original article has been updated.

