# Supplementary material for: Platelet abnormalities in autoimmune thyroid diseases: A systematic review and meta-analysis
Source: Front Immunol. 2022 Dec 22;13:1089469. doi: 10.3389/fimmu.2022.1089469 (PMC9812954; doi:10.3389/fimmu.2022.1089469)
Supplement: Supplementary file 1 [file DataSheet_1.docx]

Supplementary Material

**Supplementary Methods 1.** The Newcastle Ottawa Quality Assessment Scale - Case control study

**Supplementary Methods 2.** The Newcastle Ottawa Quality Assessment Scale - Cohort study

**Supplementary Methods 3.** The Agency for Healthcare Research and Quality methodology checklist

**Supplementary Methods 4.** Articles excluded after full text review

**Supplementary Table S1.** The Meta-analysis of Observational Studies in Epidemiology (MOOSE) reporting guideline

**Supplementary Table S2.** The Preferred Reporting Items for a Systematic Review and Meta-analysis (PRISMA) guideline

**Supplementary Table S3.** Search Strategy

**Supplementary Table S4.** Characteristics of Included Studies

**Supplementary Table S5.** Original Data and Corresponding Converted Data

**Supplementary Table S6.** Meta-regression analysis

**Supplementary Table S7.** Sensitivity analysis

**Supplementary Table S8.** Egger’s Test

**Supplementary Figure S1.** Subgroup analysis of PLT Difference between the AITD Group and the Control Group.

A disease type, B thyroid function, C quality assessment, D region, E study type

**Supplementary Figure S2.** Subgroup analysis of MPV Difference between the AITD Group and the Control Group.

A disease type, B thyroid function, C study type

**Supplementary Figure S3.** Funnel Plot

A PLT, B MPV

**Supplementary Figure S4.** Egger’s Test

A PLT, B MPV

**Supplementary Methods 1.** The Newcastle Ottawa Quality Assessment Scale - Case control study

Note: A study can be awarded a maximum of one star for each numbered item within the Selection and Exposure categories. A maximum of two stars can be given for Comparability.

Selection

1) Is the case definition adequate?

a) yes, with independent validation ☆ 

b) yes, eg record linkage or based on self reports

c) no description

2) Representativeness of the cases

a) consecutive or obviously representative series of cases ☆

b) potential for selection biases or not stated

3) Selection of Controls

a) community controls ☆ 

b) hospital controls

c) no description

4) Definition of Controls

a) no history of disease (endpoint) ☆ 

b) no description of source

Comparability

1) Comparability of cases and controls on the basis of the design or analysis

a) study controls for _______________ (Select the most important factor.) ☆ 

b) study controls for any additional factor (This criteria could be modified to indicate specific control for a second important factor.) ☆

Exposure

1) Ascertainment of exposure

a) secure record (eg surgical records) ☆ 

b) structured interview where blind to case/control status ☆ 

c) interview not blinded to case/control status

d) written self report or medical record only

e) no description

2) Same method of ascertainment for cases and controls

a) yes ☆ 

b) no

3) Non-Response rate

a) same rate for both groups ☆ 

b) non respondents described

c) rate different and no designation

**Supplementary Methods 2.** The Newcastle Ottawa Quality Assessment Scale - Cohort study

Note: A study can be awarded a maximum of one star for each numbered item within the Selection and Outcome categories. A maximum of two stars can be given for Comparability

Selection

1) Representativeness of the exposed cohort

a) truly representative of the average _______________ (describe) in the community ☆ 

b) somewhat representative of the average ______________ in the community ☆ 

c) selected group of users eg nurses, volunteers

d) no description of the derivation of the cohort

2) Selection of the non-exposed cohort

a) drawn from the same community as the exposed cohort ☆ 

b) drawn from a different source

c) no description of the derivation of the non-exposed cohort

3) Ascertainment of exposure

a) secure record (eg surgical records) ☆ 

b) structured interview ☆ 

c) written self-report

d) no description

4) Demonstration that outcome of interest was not present at start of study

a) yes ☆ 

b) no

Comparability

1) Comparability of cohorts on the basis of the design or analysis

a) study controls for _____________ (select the most important factor) ☆ 

b) study controls for any additional factor (This criteria could be modified to indicate specific control for a second important factor.) ☆

Outcome

1) Assessment of outcome

a) independent blind assessment ☆ 

b) record linkage ☆ 

c) self-report

d) no description

2) Was follow-up long enough for outcomes to occur

a) yes (select an adequate follow up period for outcome of interest) ☆ 

b) no

3) Adequacy of follow up of cohorts

a) complete follow up - all subjects accounted for ☆ 

b) subjects lost to follow up unlikely to introduce bias - small number lost - > ____ % (select an adequate %) follow up, or description provided of those lost) ☆ 

c) follow up rate < ____% (select an adequate %) and no description of those lost

d) no statement

**Supplementary Methods 3.** The Agency for Healthcare Research and Quality methodology checklist

Item

1) Define the source of information (survey, record review)

2) List inclusion and exclusion criteria for exposed and unexposed subjects (cases and controls) or refer to previous publications

3) Indicate time period used for identifying patients

4) Indicate whether or not subjects were consecutive if not population-based

5) Indicate if evaluators of subjective components of study were masked to other aspects of the status of the participants

6) Describe any assessments undertaken for quality assurance purposes (e.g., test/retest of primary outcome measurements)

7) Explain any patient exclusions from analysis

8) Describe how confounding was assessed and/or controlled.

9) If applicable, explain how missing data were handled in the analysis

10) Summarize patient response rates and completeness of data collection

11) Clarify what follow-up, if any, was expected and the percentage of patients for which incomplete data or follow-up was obtained

**Supplementary Methods 4.** Articles excluded after full text review

**A. unsuitable population**

1. Artemniak-Wojtowicz D, Witkowska-Sędek E, Borowiec A, Pyrzak B. Peripheral blood picture and aminotransferase activity in children with newly diagnosed Graves' disease at baseline and after the initiation of antithyroid drug therapy. *Cent Eur J Immunol*. 2019;44(2):132-137. doi:10.5114/ceji.2019.87063

2. Ergin Z, Savas ES, Kurnaz E, Cetinkaya S, Aycan Z. Follow-up in children with non-obese and non-autoimmune subclinical hypothyroidism. *J Pediatr Endocrinol Metab*. Oct 2018;31(10):1133-1138. doi:10.1515/jpem-2018-0095

3. Atilgan CU, Sendul SY, Kosekahya P, et al. Evaluation of Neutrophil-to-Lymphocyte Ratio and Mean Platelet Volume in Patients with Active and Inactive Thyroid Orbitopathy. *Sisli Etfal Hastan Tıp Bul*. 2018;52(1):26-30. doi:10.14744/semb.2017.07269

4. Gur EB, Karadeniz M, Inceefe H, et al. Thyroid antibodies in euthyroid and subclinical hypothyroidic pregnant women with autoimmune hypothyroidism: effects on hematological parameters and postpartum hemorrhage. *Ginekologia polska*. Sep 2015;86(9):666-671. doi:10.17772/gp/57810

5. Onbasi K, Keskin L, Ucgun S. May thyrotoxicosis trigger thrombocytopenia? Conference Abstract. *Endocr Rev*. 2014;35

6. Alcelik A, Aktas G, Eroglu M, et al. Platelet function in euthyroid patients undergoing thyroidectomy in women. *Eur Rev Med Pharmacol Sci*. Sep 2013;17(17):2350-2353.

7. Glaser NS, Styne DM. Predictors of early remission of hyperthyroidism in children. *J Clin Endocrinol Metab*. Jun 1997;82(6):1719-1726. doi:10.1210/jc.82.6.1719

8. Ford HC, Toomath RJ, Carter JM, Delahunt JW, Fagerstrom JN. Mean platelet volume is increased in hyperthyroidism. Article. *Am J Hematol*. 1988;27(3):190-193.

**B. unsuitable or no control group**

1. Öztürk M. Does suppression of tsh affect the mean platelet volume? Retrospective case control study. Article. *Eas J Med*. 2021;26(1):104-108. doi:10.5505/ejm.2021.85619

2. Ito S, Fujiwara SI, Murahashi R, et al. Clinical association between thyroid disease and immune thrombocytopenia. *Ann Hematol*. Feb 2021;100(2):345-352. doi:10.1007/s00277-020-04343-5

3. Alay M, Sönmez GM, Yildiz S. Evaluatıon of neutrophıl-to-lymphocyte ratıo and mean platelet volume in patıents with hyperthyroıdısm. Article. *Eas J Med*. 2020;25(3):388-392. doi:10.5505/ejm.2020.79037

4. Bagir GS, Haydardedeoglu FE, Bakiner OS, Bozkirli E, Ertorer ME. Mean Platelet Volume in Graves' disease: A Sign of Hypermetabolism Rather than Autoimmunity? *Pak J Med Sci*. Jul-Aug 2017;33(4):871-875. doi:10.12669/pjms.334.12659

5. van Steensel L, Paridaens D, van Meurs M, et al. Orbit-Infiltrating Mast Cells, Monocytes, and Macrophages Produce PDGF Isoforms that Orchestrate Orbital Fibroblast Activation in Graves' Ophthalmopathy. *J Clin Endocr Metab*. Mar 2012;97(3):E400-E408. doi:10.1210/jc.2011-2697

6. Li W, Guo L, Hou M. Clinical features and treatment outcomes of concurrent Graves disease and immune thrombocytopenia. *Chinese Journal of Practical Internal Medicine*. 2011;31(11):859-861. 1005-2194(2011)31:11<859:Gbhbmy>2.0.Tx;2-u.

7. Zhu TF, Yu HUA. Hematological Abnormalities in Patients with Graves' Disease and Its Correlation Factors. Graves. *Chinese General Practice*. 2010;13(8):855-856,859.

8. Ioachimescu AG, Makdissi A, Lichtin A, Zimmerman RS. Thyroid disease in patients with idiopathic thrombocytopenia: A cohort study. *Thyroid*. Nov 2007;17(11):1137-1142. doi:10.1089/thy.2007.0066

9. Panzer S, Haubenstock A, Minar E. Platelets in hyperthyroidism: studies on platelet counts, mean platelet volume, 111-indium-labeled platelet kinetics, and platelet-associated immunoglobulins G and M. *J Clin Endocrinol Metab*. Feb 1990;70(2):491-496. doi:10.1210/jcem-70-2-491

**C. unsuitable outcomes**

1. Mobarrez F, Abraham-Nordling M, Aguilera-Gatica K, et al. The expression of microvesicles in the blood of patients with Graves' disease and its relationship to treatment. *Clin Endocrinol*. May 2016;84(5):729-735. doi:10.1111/cen.12872

2. Erem C, Ersoz HO, Karti SS, et al. Blood coagulation and fibrinolysis in patients with hyperthyroidism. *J Endocrinol Invest*. Apr 2002;25(4):345-350. doi:10.1007/bf03344016

3. Hymes K, Blum M, Lackner H, Karpatkin S. Easy bruising, thrombocytopenia, and elevated platelet immunoglobulin G in Graves' disease and Hashimoto's thyroiditis. *Ann Intern Med*. Jan 1981;94(1):27-30. doi:10.7326/0003-4819-94-1-27

**D. less than 20 patients**

1. Okada M, Kamiya Y, Ito J, et al. Platelet epidermal growth factor in thyroid disorders. *Endocr J*. Feb 1998;45(1):83-88. doi:10.1507/endocrj.45.83

**E. inadequate data or no full text**

1. Gorar S, Alioglu B, Dellal FD, et al. Evaluation of Platelet Functions in Patients with Hashimoto's Thyroiditis Versus Healthy Controls: a Cross-Sectional Analysis. *Clin Lab*. 2019;65(6):953-958. doi:10.7754/Clin.Lab.2018.181009

2. Tani J, Eguchi H, Hiromatsu Y, Nomura M. Mean platelet volume is a good maker to make diagnosis of thyroid-associated ophthalmopathy and to make a prediction of its prognosis. Conference Abstract. *Thyroid*. 2017;27:A122. doi:10.1089/thy.2017.29046.abstracts

3. Bilge M, Adas M, Helvaci A. Neutrophil/lymphocyte ratio (NLR) and platelet/lymphocyte ratio (PLR) in patients with hashimoto's thyroiditis and their relationship with thyroid autoimmunity. Conference Abstract. *Endocr Rev*. 2015;36

4. Goel G, Majumdar K, Joshi D, Joshi R, Kapoor N. Role of mean platelet volume in patients with autoimmune disease. Conference Abstract. *Indian J Hematol Blo*. 2014;30(2):491-492. doi:10.1007/s12288-014-0467-0

5. Gorar S, Alioglu B, Bekdemir H, Dellal FD, Saglam B, Aral Y. Platelet aggregation and secretion tests in Hashimoto's thyroiditis. Conference Abstract. *Endocr Rev*. 2013;34(3)

6. Volpe JA, Johnston GS. Coexistent hypothyroidism and idiopathic thrombocytopenic purpura. *Mil Med*. Dec 1970;135(12):1146-1148. doi:10.1093/milmed/135.12.1146

**Supplementary Table S1.** The Meta-analysis of Observational Studies in Epidemiology (MOOSE) reporting guideline

| **Item No** | **Recommendation** | **Location where item is reported** |
| --- | --- | --- |
| **Reporting of background should include** | | |
| 1 | Problem definition | Introduction, Paragraph 3 |
| 2 | Hypothesis statement | Introduction, Paragraph 2 |
| 3 | Description of study outcome(s) | Introduction, Paragraph 3 |
| 4 | Type of exposure or intervention used | NA |
| 5 | Type of study designs used | NA |
| 6 | Study population | Introduction, Paragraph 1, 2, 3 |
| **Reporting of search strategy should include** | | |
| 7 | Qualifications of searchers (eg, librarians and investigators) | Methods, Paragraph 2 |
| 8 | Search strategy, including time period included in the synthesis and keywords | Methods, Paragraph 2, Table S3 |
| 9 | Effort to include all available studies, including contact with authors | Methods, Paragraph 5 |
| 10 | Databases and registries searched | Methods, Paragraph 1, 2 |
| 11 | Search software used, name and version, including special features used (eg, explosion) | NA |
| 12 | Use of hand searching (eg, reference lists of obtained articles) | Methods, Paragraph 2 |
| 13 | List of citations located and those excluded, including justification | Figure 1, Methods 4 |
| 14 | Method of addressing articles published in languages other than English | NA |
| 15 | Method of handling abstracts and unpublished studies | NA |
| 16 | Description of any contact with authors | Methods, Paragraph 5 |
| **Reporting of methods should include** | | |
| 17 | Description of relevance or appropriateness of studies assembled for assessing the hypothesis to be tested | NA |
| 18 | Rationale for the selection and coding of data (eg, sound clinical principles or convenience) | Methods, Paragraph 5 |
| 19 | Documentation of how data were classified and coded (eg, mutiple raters, blinding, and interrater reliability) | Methods, Paragraph 5 |
| 20 | Assessment of confounding (eg, comparability of cases and controls in studies where appropriate) | Methods, Paragraph 6 |
| 21 | Assessment of study quality, indluding blinding of quality assessors; stratifcation or regression on possible predictors of study results | Methods, Paragraph 6 |
| 22 | Assessment of heterogeneity | Methods, Paragraph 8 |
| 23 | Description of statistical methods (eg, complete description of fixed or random effects models, justification of whether the chosen models account for predictors of study results, dose-response models, or cumulative meta-analysis) in sufficient detail to be replicated | Methods, Paragraph 7, 8, 9 |
| 24 | Provision of appropriate tables and graphics | Figure 1, 2, 3, 4 |
| **Reporting of results should include** | | |
| 25 | Graphic summarizing individual study estimates and overall estimate | Figure 2 ,3, 4; Figure S1, 2 |
| 26 | Table giving descriptive information for each study included | Table S4 |
| 27 | Results of sensitivity testing (eg, subgroup analysis) | Results, Paragraph 5, 6, 7 |
| 28 | Indication of statistical uncertainty of findings | Discussion, paragraph 5 |
| **Reporting of discussion should include** | | |
| 29 | Quantitative assessment of bias (eg, publication bias) | Results, Paragraph 2, 7 |
| 30 | Justification for exclusion (eg, exclusion of non-English-language citations) | Figure 1, Methods 4 |
| 31 | Assessment of quality of included studies | Results, Paragraph 2 |
| **Reporting of conclusions should include** | | |
| 32 | Consideration of alternative explanations for observed results | Discussion, paragraph 2, 3, 4 |
| 33 | Generalization of the conclusions (ie, appropriate for the data presented and within the domain of the literature review) | Discussion, paragraph 6 |
| 34 | Guidelines for future research | Discussion, paragraph 6 |
| 35 | Disclosure of funding source | Funding |

Abbreviations: NA, not apply

**Supplementary Table S2.** The Preferred Reporting Items for a Systematic Review and Meta-analysis (PRISMA) guideline

| **Section and Topic** | **Item #** | **Checklist item** | **Location where item is reported** |
| --- | --- | --- | --- |
| **TITLE** | | |  |
| Title | 1 | Identify the report as a systematic review. | Title |
| **ABSTRACT** | | |  |
| Abstract | 2 | See the PRISMA 2020 for Abstracts checklist. | Abstract |
| **INTRODUCTION** | | |  |
| Rationale | 3 | Describe the rationale for the review in the context of existing knowledge. | Introduction,  Paragraph 1, 2 |
| Objectives | 4 | Provide an explicit statement of the objective(s) or question(s) the review addresses. | Introduction,  paragraph 3 |
| **METHODS** | | |  |
| Eligibility criteria | 5 | Specify the inclusion and exclusion criteria for the review and how studies were grouped for the syntheses. | Methods,  paragraph 3, 4, 7 |
| Information sources | 6 | Specify all databases, registers, websites, organisations, reference lists and other sources searched or consulted to identify studies. Specify the date when each source was last searched or consulted. | Methods,  paragraph 2 |
| Search strategy | 7 | Present the full search strategies for all databases, registers and websites, including any filters and limits used. | Table S3 |
| Selection process | 8 | Specify the methods used to decide whether a study met the inclusion criteria of the review, including how many reviewers screened each record and each report retrieved, whether they worked independently, and if applicable, details of automation tools used in the process. | Methods,  paragraph 5 |
| Data collection process | 9 | Specify the methods used to collect data from reports, including how many reviewers collected data from each report, whether they worked independently, any processes for obtaining or confirming data from study investigators, and if applicable, details of automation tools used in the process. | Methods,  paragraph 5 |
| Data items | 10a | List and define all outcomes for which data were sought. Specify whether all results that were compatible with each outcome domain in each study were sought (e.g. for all measures, time points, analyses), and if not, the methods used to decide which results to collect. | Methods,  paragraph 5 |
|  | 10b | List and define all other variables for which data were sought (e.g. participant and intervention characteristics, funding sources). Describe any assumptions made about any missing or unclear information. | Methods,  paragraph 5 |
| Study risk of bias assessment | 11 | Specify the methods used to assess risk of bias in the included studies, including details of the tool(s) used, how many reviewers assessed each study and whether they worked independently, and if applicable, details of automation tools used in the process. | Methods,  paragraph 6 |
| Effect measures | 12 | Specify for each outcome the effect measure(s) (e.g. risk ratio, mean difference) used in the synthesis or presentation of results. | Methods,  paragraph 7 |
| Synthesis methods | 13a | Describe the processes used to decide which studies were eligible for each synthesis (e.g. tabulating the study intervention characteristics and comparing against the planned groups for each synthesis (item #5)). | Methods,  paragraph 3, 4, 7 |
|  | 13b | Describe any methods required to prepare the data for presentation or synthesis, such as handling of missing summary statistics, or data conversions. | Methods,  paragraph 7 |
|  | 13c | Describe any methods used to tabulate or visually display results of individual studies and syntheses. | Methods,  paragraph 7 |
|  | 13d | Describe any methods used to synthesize results and provide a rationale for the choice(s). If meta-analysis was performed, describe the model(s), method(s) to identify the presence and extent of statistical heterogeneity, and software package(s) used. | Methods,  paragraph 7, 8, 9, 10 |
|  | 13e | Describe any methods used to explore possible causes of heterogeneity among study results (e.g. subgroup analysis, meta-regression). | Methods,  paragraph 8 |
|  | 13f | Describe any sensitivity analyses conducted to assess robustness of the synthesized results. | Methods,  paragraph 9 |
| Reporting bias assessment | 14 | Describe any methods used to assess risk of bias due to missing results in a synthesis (arising from reporting biases). | NA |
| Certainty assessment | 15 | Describe any methods used to assess certainty (or confidence) in the body of evidence for an outcome. | Methods,  paragraph 7 |
| **RESULTS** | | |  |
| Study selection | 16a | Describe the results of the search and selection process, from the number of records identified in the search to the number of studies included in the review, ideally using a flow diagram. | Results,  paragraph 1  Figure 1 |
|  | 16b | Cite studies that might appear to meet the inclusion criteria, but which were excluded, and explain why they were excluded. | Figure 1,  Methods 4 |
| Study characteristics | 17 | Cite each included study and present its characteristics. | Results,  paragraph 2,  Table S4 |
| Risk of bias in studies | 18 | Present assessments of risk of bias for each included study. | Results,  paragraph 2,  Table S4 |
| Results of individual studies | 19 | For all outcomes, present, for each study: (a) summary statistics for each group (where appropriate) and (b) an effect estimate and its precision (e.g. confidence/credible interval), ideally using structured tables or plots. | Results,  paragraph 3, 5, 6  Figure 2, 3, 4  Figure S1, 2 |
| Results of syntheses | 20a | For each synthesis, briefly summarise the characteristics and risk of bias among contributing studies. | Results,  paragraph 2,  Table S4 |
|  | 20b | Present results of all statistical syntheses conducted. If meta-analysis was done, present for each the summary estimate and its precision (e.g. confidence/credible interval) and measures of statistical heterogeneity. If comparing groups, describe the direction of the effect. | Results,  paragraph 3, 5, 6 |
|  | 20c | Present results of all investigations of possible causes of heterogeneity among study results. | Results,  paragraph 4 |
|  | 20d | Present results of all sensitivity analyses conducted to assess the robustness of the synthesized results. | Results,  paragraph 7 |
| Reporting biases | 21 | Present assessments of risk of bias due to missing results (arising from reporting biases) for each synthesis assessed. | NA |
| Certainty of evidence | 22 | Present assessments of certainty (or confidence) in the body of evidence for each outcome assessed. | Results,  paragraph 3, 5, 6  Figure 2, 3, 4  Figure S1, 2 |
| **DISCUSSION** | | |  |
| Discussion | 23a | Provide a general interpretation of the results in the context of other evidence. | Discussion,  paragraph 1, 2, 3, 4 |
|  | 23b | Discuss any limitations of the evidence included in the review. | Discussion,  paragraph 5 |
|  | 23c | Discuss any limitations of the review processes used. | Discussion,  paragraph 5 |
|  | 23d | Discuss implications of the results for practice, policy, and future research. | Discussion,  paragraph 6 |
| **OTHER INFORMATION** | | |  |
| Registration and protocol | 24a | Provide registration information for the review, including register name and registration number, or state that the review was not registered. | Methods,  paragraph 1 |
|  | 24b | Indicate where the review protocol can be accessed, or state that a protocol was not prepared. | Methods,  paragraph 1 |
|  | 24c | Describe and explain any amendments to information provided at registration or in the protocol. | NA |
| Support | 25 | Describe sources of financial or non-financial support for the review, and the role of the funders or sponsors in the review. | Funding |
| Competing interests | 26 | Declare any competing interests of review authors. | Conflict of Interest |
| Availability of data, code and other materials | 27 | Report which of the following are publicly available and where they can be found: template data collection forms; data extracted from included studies; data used for all analyses; analytic code; any other materials used in the review. | Data availability statement |

Abbreviations: NA, not apply

**Supplementary Table S3.** Search Strategy

| **Database** | **Search strategy** | | **Results** |
| --- | --- | --- | --- |
| **1)PubMed**  **(To August 16, 2022)** | #1 | "Graves disease"[MeSH Terms] OR "Hashimoto disease"[MeSH Terms] OR "autoimmune thyroid disorder*"[Title/Abstract] OR "autoimmune thyroid disease*"[Title/Abstract] | 24400 |
|  | #2 | Disease*, Graves[Title/Abstract] OR Basedow Disease*[Title/Abstract] OR Disease*, Basedow[Title/Abstract] OR Graves' Disease*[Title/Abstract] OR Disease*, Graves'[Title/Abstract] OR Exophthalmic Goiter*[Title/Abstract] OR Goiter*, Exophthalmic[Title/Abstract] OR Hyperthyroidism, Autoimmune [Title/Abstract] OR Basedow's Disease*[Title/Abstract] OR Basedows Disease*[Title/Abstract] OR Disease*, Basedow's[Title/Abstract] OR Disease*, Hashimoto[Title/Abstract] OR Hashimoto Struma[Title/Abstract] OR Hashimoto Thyroiditi*[Title/Abstract] OR Thyroiditi*, Hashimoto[Title/Abstract] OR Hashimoto's Syndrome*[Title/Abstract] OR Hashimoto Syndrome*[Title/Abstract] OR Hashimotos Syndrome*[Title/Abstract] OR Syndrome*, Hashimoto's[Title/Abstract] OR Hashimoto's Struma[Title/Abstract] OR Chronic Lymphocytic Thyroiditi*[Title/Abstract] OR Lymphocytic Thyroiditi*, Chronic[Title/Abstract] OR Thyroiditi*, Chronic Lymphocytic[Title/Abstract] OR Hashimoto's Disease*[Title/Abstract] OR Disease*, Hashimoto's[Title/Abstract] OR Hashimotos Disease*[Title/Abstract] | 25051 |
|  | #3 | #1 OR #2 | 33960 |
|  | #4 | " Mean Platelet Volume "[MeSH Terms] OR Platelet Count [MeSH Terms] OR platelet distribution width[Title/Abstract] | 25010 |
|  | #5 | Mean Platelet Volume*[Title/Abstract] OR Platelet Volume*, Mean[Title/Abstract] OR Volume*, Mean Platelet[Title/Abstract] OR MPV[Title/Abstract] OR Count*, Platelet[Title/Abstract] OR Platelet Count*[Title/Abstract] OR Platelet Number*[Title/Abstract] OR Number*, Platelet[Title/Abstract] OR Blood Platelet Number*[Title/Abstract] OR Number*, Blood Platelet[Title/Abstract] OR Platelet Number*, Blood[Title/Abstract] OR Blood Platelet Count*[Title/Abstract] OR Count*, Blood Platelet[Title/Abstract] OR Platelet Count*, Blood[Title/Abstract] OR PLT[Title/Abstract] OR PDW[Title/Abstract] | 34675 |
|  | #6 | #4 OR #5 | 49857 |
|  | #7 | #3 AND #6 | 74 |
| **2) Embase**  **(To August 16, 2022)** | #1 | 'graves disease'/exp OR 'hashimoto disease'/exp | 36380 |
|  | #2 | 'autoimmune thyroid disorder*':ti,ab,kw OR 'autoimmune thyroid disease*':ti,ab,kw OR 'basedow disease*':ti,ab,kw OR 'disease*, basedow':ti,ab,kw OR 'graves disease*':ti,ab,kw OR 'disease*, graves':ti,ab,kw OR 'exophthalmic goiter*':ti,ab,kw OR 'goiter*, exophthalmic':ti,ab,kw OR 'hyperthyroidism, autoimmune':ti,ab,kw OR 'basedows disease*':ti,ab,kw OR 'disease*, basedows':ti,ab,kw OR 'disease*, hashimoto':ti,ab,kw OR 'hashimoto struma':ti,ab,kw OR 'hashimoto thyroiditi*':ti,ab,kw OR 'thyroiditi*, hashimoto':ti,ab,kw OR 'hashimoto syndrome*':ti,ab,kw OR 'hashimotos syndrome*':ti,ab,kw OR 'syndrome*, hashimotos':ti,ab,kw OR 'hashimotos struma':ti,ab,kw OR 'chronic lymphocytic thyroiditi*':ti,ab,kw OR 'lymphocytic thyroiditi*, chronic':ti,ab,kw OR 'thyroiditi*, chronic lymphocytic':ti,ab,kw OR 'hashimotos disease*':ti,ab,kw OR 'disease*, hashimotosor hashimotos disease*':ti,ab,kw | 24017 |
|  | #3 | #1 OR #2 | 42346 |
|  | #4 | 'mean platelet volume'/exp OR 'platelet count'/exp OR 'platelet distribution width'/exp | 115371 |
|  | #5 | 'mean platelet volume*or platelet volume*, mean':ti,ab,kw OR 'volume*, mean platelet':ti,ab,kw OR mpv:ti,ab,kw OR 'count*, platelet':ti,ab,kw OR 'platelet count*':ti,ab,kw OR 'platelet number*':ti,ab,kw OR 'number*, platelet':ti,ab,kw OR 'blood platelet number*':ti,ab,kw OR 'number*, blood platelet':ti,ab,kw OR 'platelet number*, blood':ti,ab,kw OR 'blood platelet count*':ti,ab,kw OR 'count*, blood platelet':ti,ab,kw OR 'platelet count*, blood':ti,ab,kw OR plt:ti,ab,kw OR pdw:ti,ab,kw | 83298 |
|  | #6 | #4 OR #5 | 142034 |
|  | #7 | #3 AND #6 | 267 |
| **3) Cochrane Library**  **(To August 16, 2022)** | #1 | MeSH descriptor: [Graves Disease] explode all trees OR MeSH descriptor: [Hashimoto Disease] explode all trees | 526 |
|  | #2 | (autoimmune thyroid disorder* OR autoimmune thyroid disease* OR Disease*, Graves OR Basedow Disease* OR Disease*, Basedow OR Graves' Disease* OR Disease*, Graves' OR Exophthalmic Goiter* OR Goiter*, Exophthalmic OR Hyperthyroidism, Autoimmune OR Basedow's Disease* OR Basedows Disease* OR Disease*, Basedow's OR Disease*, Hashimoto OR Hashimoto Struma OR Hashimoto Thyroiditi* OR Thyroiditi*, Hashimoto OR Hashimoto's Syndrome* OR Hashimoto Syndrome* OR Hashimotos Syndrome* OR Syndrome*, Hashimoto's OR Hashimoto's Struma OR Chronic Lymphocytic Thyroiditi* OR Lymphocytic Thyroiditi*, Chronic OR Thyroiditi*, Chronic Lymphocytic OR Hashimoto's Disease* OR Disease*, Hashimoto'sOR Hashimotos Disease*):ti,ab,kw | 3144 |
|  | #3 | #1 or #2 | 3163 |
|  | #4 | MeSH descriptor: [Mean Platelet Volume] explode all trees OR MeSH descriptor: [Platelet Count] explode all trees | 1293 |
|  | #5 | (platelet distribution width OR Mean Platelet Volume*OR Platelet Volume*, Mean OR Volume*, Mean Platelet OR MPV OR Count*, Platelet OR Platelet Count* OR Platelet Number* OR Number*, Platelet OR Blood Platelet Number* OR Number*, Blood Platelet OR Platelet Number*, Blood OR Blood Platelet Count* OR Count*, Blood Platelet OR Platelet Count*, Blood OR PLT OR PDW):ti,ab,kw | 13425 |
|  | #6 | #4 OR #5 | 13426 |
|  | #7 | #3 AND #6 | 51 |
| **4) Web of science**  **(To August 16, 2022)** | #1 | TS= (autoimmune thyroid disorder* OR autoimmune thyroid disease* OR Graves disease* OR Hashimoto disease* OR Basedow Disease* OR Exophthalmic Goiter* OR Hyperthyroidism, Autoimmune OR Hashimoto Struma OR Hashimoto Thyroiditi* OR Hashimoto's Syndrome* OR Chronic Lymphocytic Thyroiditi* OR Lymphocytic Thyroiditi*) | 65720 |
|  | #2 | TS= (Mean Platelet Volume* OR Platelet Volume*, Mean OR Volume*, Mean Platelet OR MPV OR Platelet Count* OR Count*, Platelet OR Platelet Number* OR Number*, Platelet OR Blood Platelet Number* OR Number*, Blood Platelet OR Platelet Number*, Blood OR Blood Platelet Count* OR Count*, Blood Platelet OR Platelet Count*, Blood OR PLT OR platelet distribution width OR PDW) | 140612 |
|  | #3 | #1 AND #2 | 367 |

**Supplementary Table S4.** Characteristics of Included Studies

| **Source** | **Study type** | **Disease type** | **Case group** | | | | | | **Control group** | | | | | | **Hematology Analyzer** | **NOS** | **AHRQ Score** |
| --- | --- | --- | --- | --- | --- | --- | --- | --- | --- | --- | --- | --- | --- | --- | --- | --- | --- |
|  |  |  | Sample (n) | Gender (M/F) | Age (year) | PLT (10^9^/L) | MPV (f/L) | PDW (%) | Sample (n) | Gender (M/F) | Age (year) | PLT (10^9^/L) | MPV (f/L) | PDW (%) |  |  |  |
| He P et al, 2022, China (23) | Case control study | GD | 146 | 109/37 | 40.1±12.0^b^ | 263.47±59.39 | 9.39±1.40 | NA | 100 | 78/22 | 42.0±14.5^b^ | 258.51±58.1 | 9.24±1.27 | NA | Coulter LH780 | 8 | NA |
| Turan E, 2019, Turkey (29) | Case control study | GD | 86 | 61/25 | 44.5±12.9 | 288.31±66.19 | NA | NA | 112 | 75/37 | 43.1±12.7 | 275±64.3 | NA | NA | NA | 7 | NA |
|  |  | GD (before treatment) | 37 | 9/28 | 43.40±13.2 | 306.02±64.03 | NA | NA |  |  |  |  |  |  |  |  |  |
|  |  | GD (after treatment) | 49 | 16/33 | 45.34±12.77 | 268.1±72.2 | NA | NA |  |  |  |  |  |  |  |  |  |
| Taşkaldiran I et al, 2019, Turkey (30) | Case control study | GD (before treatment) | 66 | 48/18 | 37.53±11.68 | 270.38±53.45^a^ | 10.39±1.24 | NA | 35 | 26/9 | 45.37±8.06 | 270.68±52.07^a^ | 9.76±1.06 | NA | NA | 7 | NA |
| Sit M et al, 2014, Turkey (37) | Case control study | HT | 97 | 18/79 | 36.8±10.0^a^ | 282 ± 62 | 8.89±0.86^a^ | 13.66±3.52^a^ | 65 | 8/57 | 39.8±7.9^a^ | 282 ±60 | 9.41±1.00^a^ | 13.77±1.66^a^ | Coulter LH780 | 8 | NA |
| Onalan E et al, 2020, Turkey (26, 27) | Cross sectional study | HT | 377 | 38/339 | 45.3±13.8 | 289.97±86.95 | NA | NA | 100 | 44/56 | 36.6±10.7 | 262.02±55.18 | NA | NA | NA | NA | 8 |
|  |  | HT (hypothyroid or subclinical hypothyroid) | 121 | 18/103 | 44.5±14.5 | 270.98±71.70 | NA | NA | 100 | 44/56 | 36.6±10.8 | 262.02±55.18 | NA | NA | NA | NA | 8 |
| Bilge M er al, 2019, Turkey (31) | Cross sectional study | HT (euthyroid) | 145 | 0/145 | 42.74±11.14 | 288.51±61.83 | NA | NA | 60 | 0/60 | 42.83±9.97 | 241.04 ± 40.94 | NA | NA | Sysmex XE-2100 | NA | 9 |
| Carlioglu A et al, 2015, Turkey (9) | Case control study | HT (euthyroid without treatment) | 51 | 9/42 | 33.88±12.87 | 263.78±67.16 | 8.8±1.05 | NA | 51 | 27/26 | 30.18±12.43 | 256.08±54.76 | 7.9±0.79 | NA | NA | 5 | NA |
| Szydelko J et al, 2020, Poland (25) | Cross sectional study | GD with GO | 168 | 18/82 | 50.4±12.9 | 252.88±65.07 | 8.21±1.13 | NA | 100 | 35/65 | 42.5±17.3 | 248.57±64.36 | 7.87±0.89 | NA | NA | NA | 9 |
|  |  | GD without GO | 238 | 19/81 | 46.7±16.5 | 257.57±70.42 | 8.11±1.04 | NA |  |  |  |  |  |  |  |  |  |
| Dasgupta R et al, 2020, India (28) | Cross sectional study | GD (before treatment) | 354 | 145/209 | 47.6±11.6 | 219±74 | NA | NA | 250 | 100/150 | 44.3±12.5 | 262±84 | NA | NA | NA | NA | 10 |
| Kuznik BI et al, 2014, Russia (5) | Case control study | GD (before treatment) | 150 | 49/101 | 31.6±12.7 | 232±89 | NA | NA | 150 | 50/100 | 33.1±10.9 | 262±95 | NA | NA | Cell-Dyn 3000 | 8 | NA |
| Keskin H et al, 2016, Turkey (35) | Case control study | HT (euthyroid) | 59 | 9/50 | 33.9±12.9 | 263.8±671 | 8.8±1.0 | NA | 53 | 27/26 | 30.2±12.4 | 256.1±54.7 | 7.9±0.8 | NA | Sysmex XE 2100 | 8 | NA |
| Demir AD, 2021, Turkey (24) | Case control study | HT | 67 | 9/54 | NA | 264.456±87.696 | NA | 12.54±1.930 | 17 | 4/13 | NA | 259.025±91.975 | NA | 12.44± 2.335 | Sysmex XN 1000 | 8 | NA |
| Atile NS et al, 2012, Turkey (39) | Case control study | HT (hypothyroid/before treatment) | 30 | NA | 46.2±12.6 | 284.0±87.0 | 9.10±1.22 | 16.9±1.0 | 20 | NA | 43.2±9.1 | 249.0±58.0 | 7.92±0.83 | 16.6±0.9 | Coulter LH 780 | 7 | NA |
|  |  | HT (euthyroid/after treatment) | 30 | NA | 46.2±12.6 | 293.7±96.9 | 8.70±1.0 | 16.76±1.29 |  |  |  |  |  |  |  |  |  |
| Aktas G et al, 2014, Turkey (38) | Case control study | HT | 102 | 17/85 | 36.8±12.1 | 279.65±66.25^a^ | NA | NA | 63 | 8/55 | 38.6±8.8 | 281.61±70.36^a^ | NA | NA | NA | 6 | NA |
| Aktas G et al, 2017, Turkey (33) | Case control study | HT | 90 | 15/75 | 37.3±11.5 | 299±63 | NA | NA | 64 | 8/56 | 39.2±9 | 301±63 | NA | NA | Coulter LH 780 | 7 | NA |
| Savas E, et al, 2016, Turkey (34) | Cross sectional study | AITD | 76 | NA | NA | NA | 8.96±1.07 | NA | 59 | 4/55 | NA | NA | 9.08±1.25 | NA | Beckman Coulter | NA | 9 |
| Arpaci D et al, 2016, Turkey (36) | Cross sectional study | HT | 92 | 5/87 | 33.12±7.33 | 291.61±81.15 | 7.64±1.39 | NA | 38 | 3/35 | 31.84±5.99 | 251.83±53.29 | 7.83±1.13 | NA | Cell-Dyn 3700 SL | NA | 10 |
|  |  | HT (euthyroid) | 42 | NA | NA | 278.17±74.65 | 7.86±1.19 | NA |  |  |  |  |  |  |  |  |  |
|  |  | HT (subclinical hypothyroid) | 38 | NA | NA | 290.22±82.61 | 7.74±1.54 | NA |  |  |  |  |  |  |  |  |  |
|  |  | HT (hypothyroid) | 12 | NA | NA | 291.62±90.26 | 7.33±0.71 | NA |  |  |  |  |  |  |  |  |  |
| Gu et al, 2017, China (32) | Cross sectional study | GD (hyperthyroid) | 978 | 313/665 | 46.28±10.66 | 232±57.2 | NA | NA | 1012 | 323/689 | 46.80±9.75 | 222±52.2 | NA | NA | UniCel DxH 800 | NA | 10 |
|  |  | HT (hyperthyroid) | 452 | 143/309 | 46.11±10.66 | 238±84.6 | NA | NA |  |  |  |  |  |  |  |  |  |

Abbreviations: GD, Graves’ disease; HT, Hashimoto’s thyroiditis; AITD, autoimmune thyroid disease; GO: Graves' Orbitopathy; M, male; F, Female; PLT, platelet count; MPV, mean platelet volume; PDW, platelet distribution width; NA, not applicable; NOS: Newcastle-Ottawa Scale; AHRQ: Agency for Healthcare Research and Quality

Values are presented as mean±SD;

^a^: Converted value, the original value was presented in median (range)

^b^: Converted value, the original value was presented in median (25–75th percentile)

**Supplementary Table S5.** Original Data and Corresponding Converted Data

| **Source** | **Variable** | **Group** | **Original Data** | **Converted Data** |
| --- | --- | --- | --- | --- |
| He P et al, 2022, China (23) | Age (year) | Case group | 38 (33-49)^b^ | 40.1±12.0 |
|  |  | Control group | 40.5 (33-52.25)^b^ | 42.0±14.5 |
| Taşkaldiran I et al, 2019, Turkey (30) | PLT (10^9^/L) | Case group | 265 (176–427)^a^ | 270.38±53.45 |
|  |  | Control group | 266 (178–397)^a^ | 270.68±52.07 |
| Sit M et al, 2014, Turkey (37) | Age (year) | Case group | 36 (18-68)^a^ | 36.8±10.0 |
|  |  | Control group | 40 (20-57)^a^ | 39.8±7.9 |
|  | MPV (f/L) | Case group | 8.9 (6.7-11)^a^ | 8.89±0.86 |
|  |  | Control group | 9.3 (7.7-12.4)^a^ | 9.41±1.00 |
|  | PDW (%) | Case group | 13.4 (6.9-24.4)^a^ | 13.66±3.52 |
|  |  | Control group | 13.5 (11.4-19.2)^a^ | 13.77±1.66 |
| Aktas G et al, 2014, Turkey (38) | PLT (10^9^/L) | Case group | 272 (175–507)^a^ | 279.65±66.25 |
|  |  | Control group | 276 (149–477)^a^ | 281.61±70.36 |

Abbreviations: PLT, platelet count; MPV, mean platelet volume; PDW, platelet distribution width

^a^: The original value was presented in median (range)

^b^: The original value was presented in median (25–75th percentile)

**Supplementary Table S6.** Meta-regression analysis

|  | **Variable** | **Coeff.** | **Std. Err.** | **z** | **P>\|z\|** | **95%CI** |
| --- | --- | --- | --- | --- | --- | --- |
| **PLT** |  |  |  |  |  |  |
|  | Disease type | 0.2467942 | 0.1424467 | 1.73 | 0.083 | (-0.0323961, 0.5259846) |
|  | Thyroid function | -0.0187647 | 0.049015 | -0.38 | 0.702 | (-0.1148324, 0.077303) |
|  | Quality assessment | -0.2108164 | 0.2419071 | -0.87 | 0.383 | (-0.6849456, 0.2633127) |
|  | Region | 0.0744257 | 0.0954426 | 0.78 | 0.436 | (-0.1126384, 0.2614898) |
|  | Study type | 0.1426405 | 0.1332748 | 1.07 | 0.284 | (-0.1185733, 0.4038543) |
| **MPV** | Disease type | -0.344017 | 0.4294242 | -0.80 | 0.423 | (-1.185673, 0.497639) |
|  | Thyroid function | -0.23465 | 0.1361666 | -1.72 | 0.085 | (-0.5015318, 0.0322317) |
|  | Quality assessment | 0.278134 | 0.5364191 | 0.52 | 0.604 | (-0.773228, 1.329496) |
|  | Region | -0.0060979 | 0.3443214 | -0.02 | 0.986 | (-0.6809555, 0.6687597) |
|  | Study type | -0.2838084 | 0.2828916 | -1.00 | 0.316 | (-0.8382658, 0.270649) |

Abbreviations: PLT, platelet count; MPV, mean platelet volume; 95% CI: 95% confidence interval

**Supplementary Table S7.** Sensitivity analysis

|  | **Study omitted** | **Estimate** | **95%CI** |
| --- | --- | --- | --- |
| **PLT** |  |  |  |
|  | He P et al, 2022 (23^)^ | 0.16900516 | (0.04345078, 0.29455954) |
|  | Turan E, 2019 ^(^29) | 0.16337477 | (0.03838039, 0.28836915) |
|  | Turan E, 2019^a^ (29) | 0.15244606 | (0.02991596, 0.27497616) |
|  | Turan E, 2019^b^ (29) | 0.1753978 | (0.05175086, 0.29904473) |
|  | Taşkaldiran I et al, 2019 (30) | 0.17069624 | (0.047123, 0.29426947) |
|  | Sit M et al, 2014 (37) | 0.17180015 | (0.04739407, 0.29620624) |
|  | Onalan E et al, 2020 (27) | 0.15622713 | (0.03153944, 0.28091481) |
|  | Onalan E et al, 2020^a^ (26) | 0.16645001 | (0.04105358, 0.29184642) |
|  | Bilge M er al, 2019 (31) | 0.13277842 | (0.01715087, 0.24840596) |
|  | Carlioglu A et al, 2015 (9) | 0.16628154 | (0.04236851, 0.29019457) |
|  | Szydelko J et al, 2020 (25) | 0.16989328 | (0.04425167, 0.29553491) |
|  | Szydelko J et al, 2020^a^ (25) | 0.16706887 | (0.04102286, 0.2931149) |
|  | Dasgupta R et al, 2020(28^)^ | 0.19052055 | (0.09858878, 0.28245232) |
|  | Kuznik BI et al, 2014 (5) | 0.18634886 | (0.06709753, 0.30560017) |
|  | Keskin H et al, 2016 (35) | 0.16638282 | (0.04231878, 0.29044688) |
|  | Demir AD, 2021 (24) | 0.16752042 | (0.04455354, 0.29048732) |
|  | Atile NS et al, 2012 (39) | 0.15713942 | (0.03487262, 0.27940622) |
|  | Atile NS et al, 2012^a^ (39) | 0.15511046 | (0.0331349, 0.27708602) |
|  | Aktas G et al, 2014 (38) | 0.17294867 | (0.04867828, 0.29721907) |
|  | Aktas G et al, 2017 (33) | 0.17295471 | (0.04876082, 0.29714859) |
|  | Arpaci D et al, 2016 (36) | 0.15050086 | (0.02844106, 0.27256066) |
|  | Arpaci D et al, 2016^a^ (36) | 0.15675989 | (0.03389732, 0.27962247) |
|  | Arpaci D et al, 2016^b^ (36) | 0.1519545 | (0.02997104, 0.27393797) |
|  | Arpaci D et al, 2016^c^ (36) | 0.15454234 | (0.03299259, 0.27609208) |
|  | Gu et al, 2017 (32) | 0.16853766 | (0.03134844, 0.30572689) |
|  | Gu et al, 2017^a^ (32) | 0.16282582 | (0.03152308, 0.29412857) |
| **MPV** |  |  |  |
|  | He P et al, 2022 (23) | 0.27086183 | (-0.00078864, 0.5425123) |
|  | Taşkaldiran I et al, 2019 (30) | 0.23559794 | (-.002129078, 0.49248666) |
|  | Sit M et al, 2014 (37) | 0.32237181 | (0.09865461, 0.54608899) |
|  | Carlioglu A et al, 2015 (9) | 0.19979934 | (-0.03812463, 0.43772331) |
|  | Szydelko J et al, 2020 (25) | 0.25216389 | (-0.01970164, 0.52402943) |
|  | Szydelko J et al, 2020^a^ (25) | 0.25989619 | (-0.0156453, 0.53543764) |
|  | Keskin H et al, 2016 (35) | 0.19669494 | (-0.03817632, 0.43156621) |
|  | Atile NS et al, 2012 (39) | 0.20512225 | (-0.03709065, 0.44733512) |
|  | Atile NS et al, 2012^a^ (39)^,^ | 0.22021565 | (-0.02868168, 0.46911299) |
|  | Savas E, et al, 2016 (34) | 0.28675368 | (0.02775563, 0.54575175) |
|  | Arpaci D et al, 2016 (36) | 0.28868836 | (0.03189468, 0.54548204) |
|  | Arpaci D et al, 2016^a^ (36) | 0.274331 | (0.01548059, 0.53318143) |
|  | Arpaci D et al, 2016^b^ (36) | 0.28080302 | (0.02362353, 0.53798252) |
|  | Arpaci D et al, 2016^c^ (36) | 0.29806188 | (0.05015384, 0.5459699) |

Abbreviations: PLT, platelet count; MPV, mean platelet volume; 95% CI: 95% confidence interval

^a^: the second available data from the same study

^b^: the third available data from the same study

^c^: the fourth available data from the same study

**Supplementary Table S8.** Egger’s Test

|  | **Std_Eff** | **Coef.** | **Std. Err.** | **t** | **P>\|t\|** | **95% Conf. Interval** |
| --- | --- | --- | --- | --- | --- | --- |
| **PLT** | slope | 0.0655675 | 0.1080028 | 0.61 | 0.549 | -0.1573394, 0.2884744 |
|  | bias | 0.5820367 | 0.8977198 | 0.65 | 0.523 | -1.270766, 2.434839 |
| **MPV** | slope | -0.0653241 | 0.4057883 | -0.16 | 0.875 | -0.9494608, 0.8188126 |
|  | bias | 1.589116 | 2.243142 | 0.71 | 0.492 | -3.298271, 6.476503 |

Abbreviations: PLT, platelet count; MPV, mean platelet volume

**Supplementary Figure S1.** Subgroup analysis of PLT Difference between the AITD Group and the Control Group.

A disease type B thyroid function


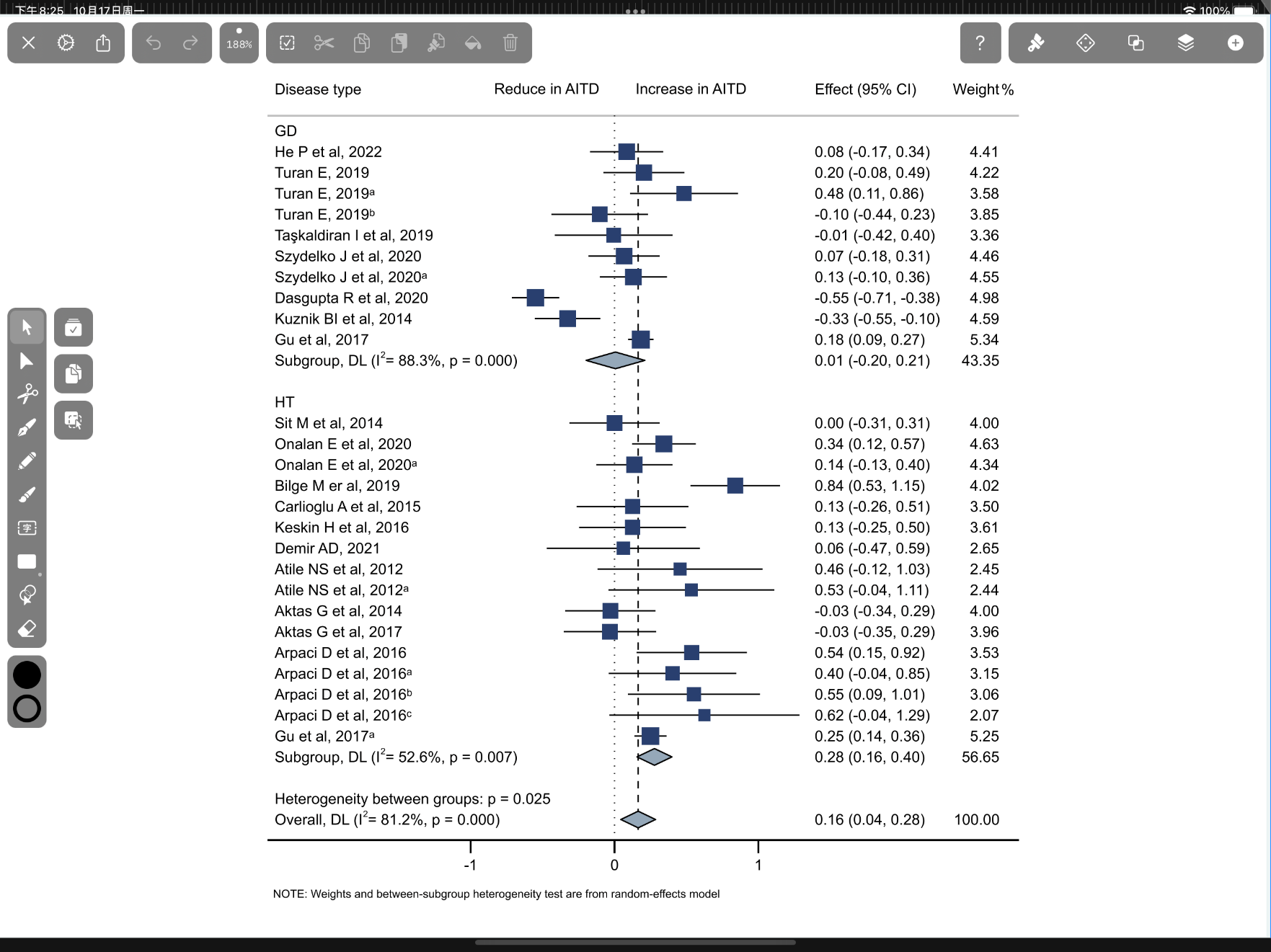

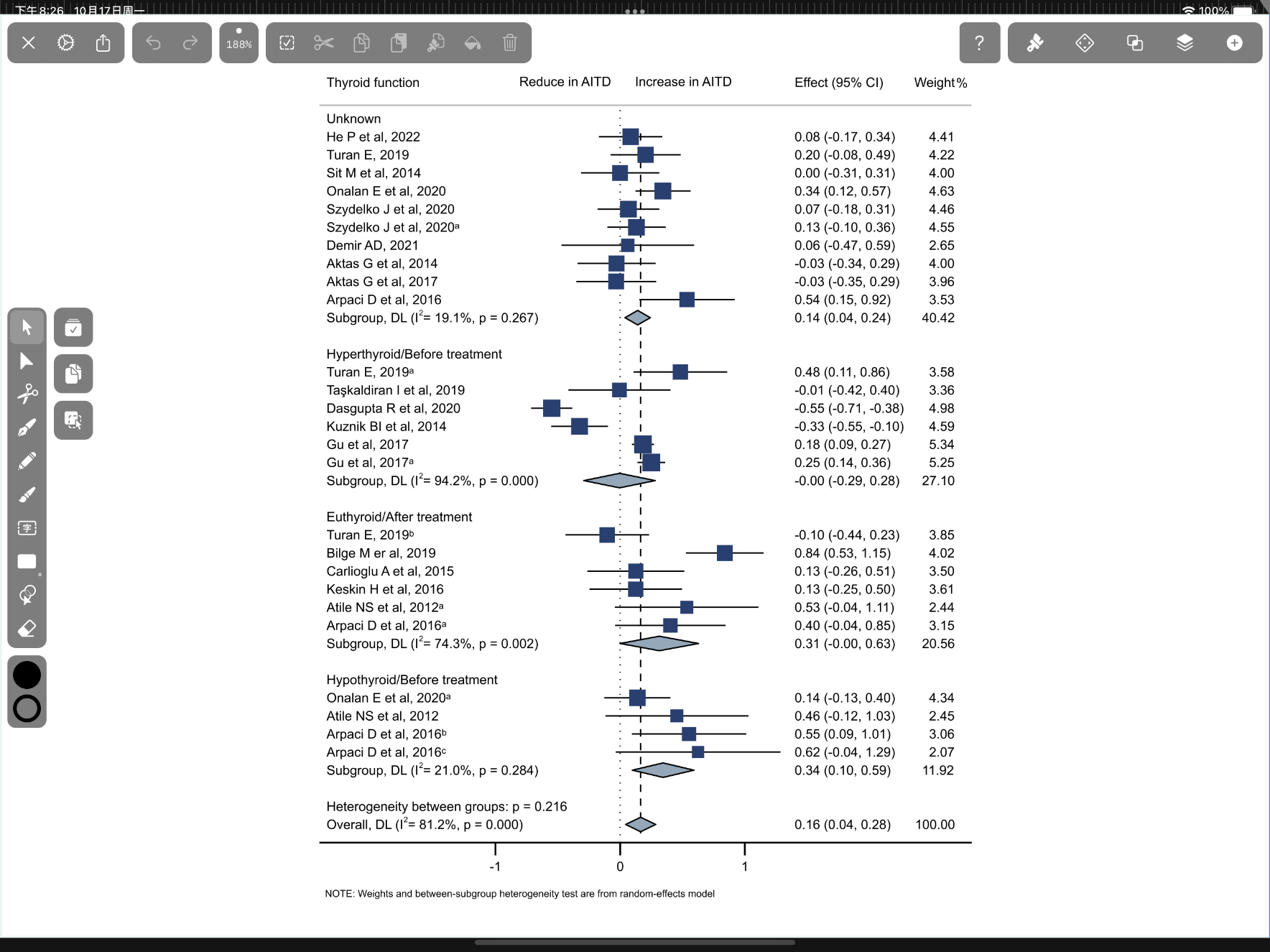


C quality assessment D region


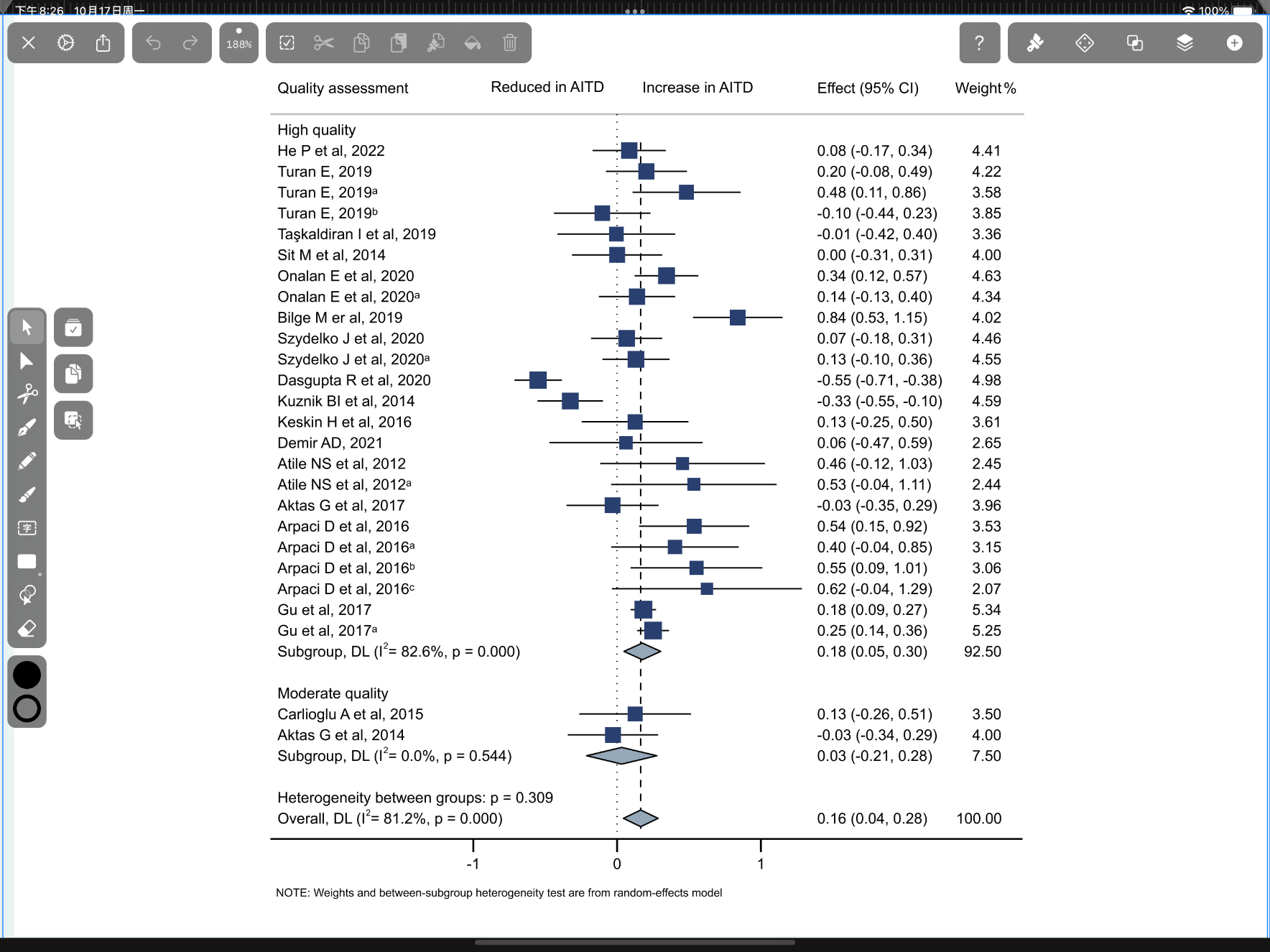

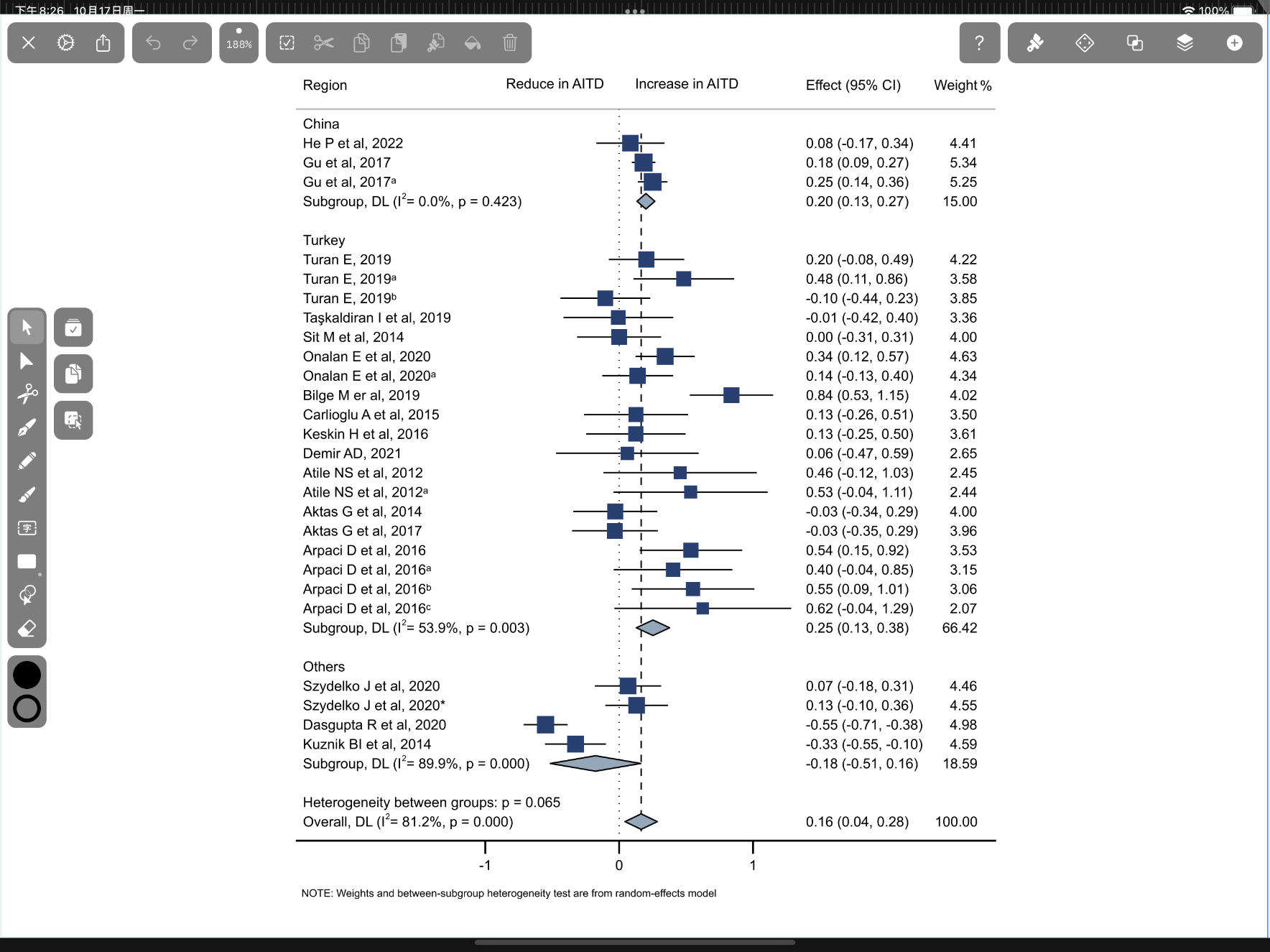


E study type

**
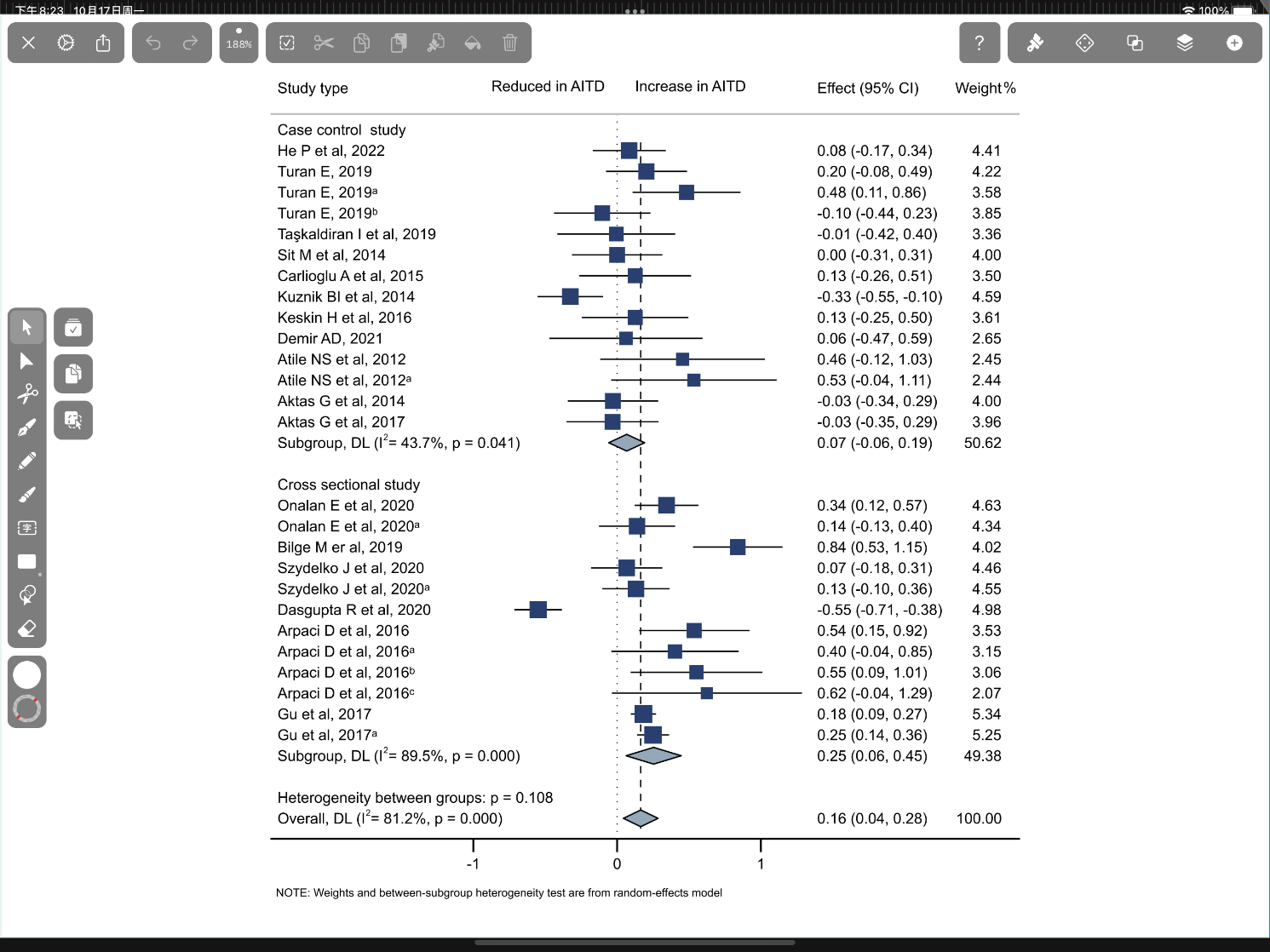
**

Abbreviations: GD, Graves’ disease; HT, Hashimoto’s thyroiditis; AITD, autoimmune thyroid disease; PLT, platelet count; 95% CI: 95% confidence interval

^a^: the second available data from the same study

^b^: the third available data from the same study

^c^: the fourth available data from the same study

**Supplementary Figure S2.** Subgroup analysis of MPV Difference between the AITD Group and the Control Group.

A disease type

**
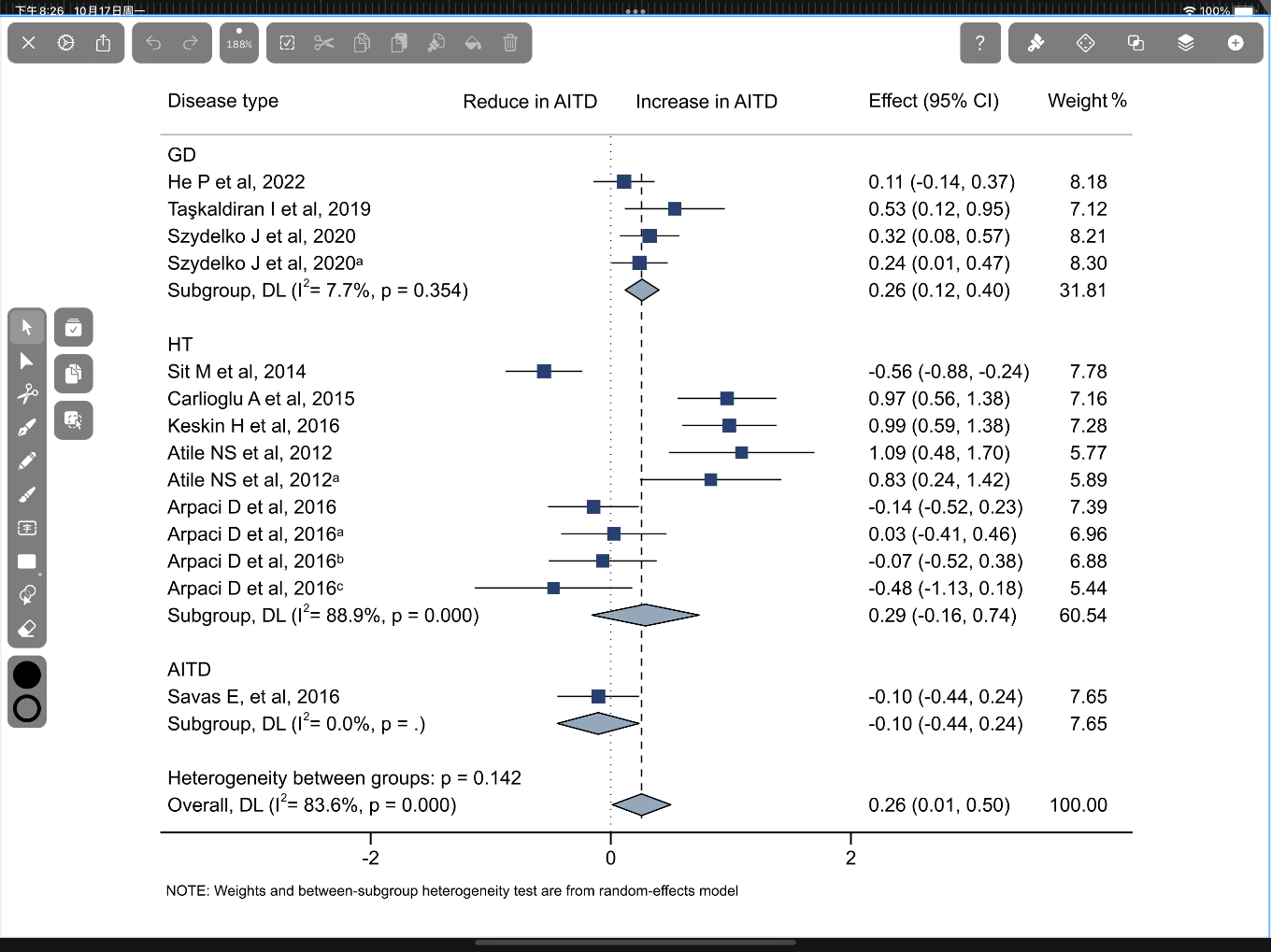
**

B thyroid function


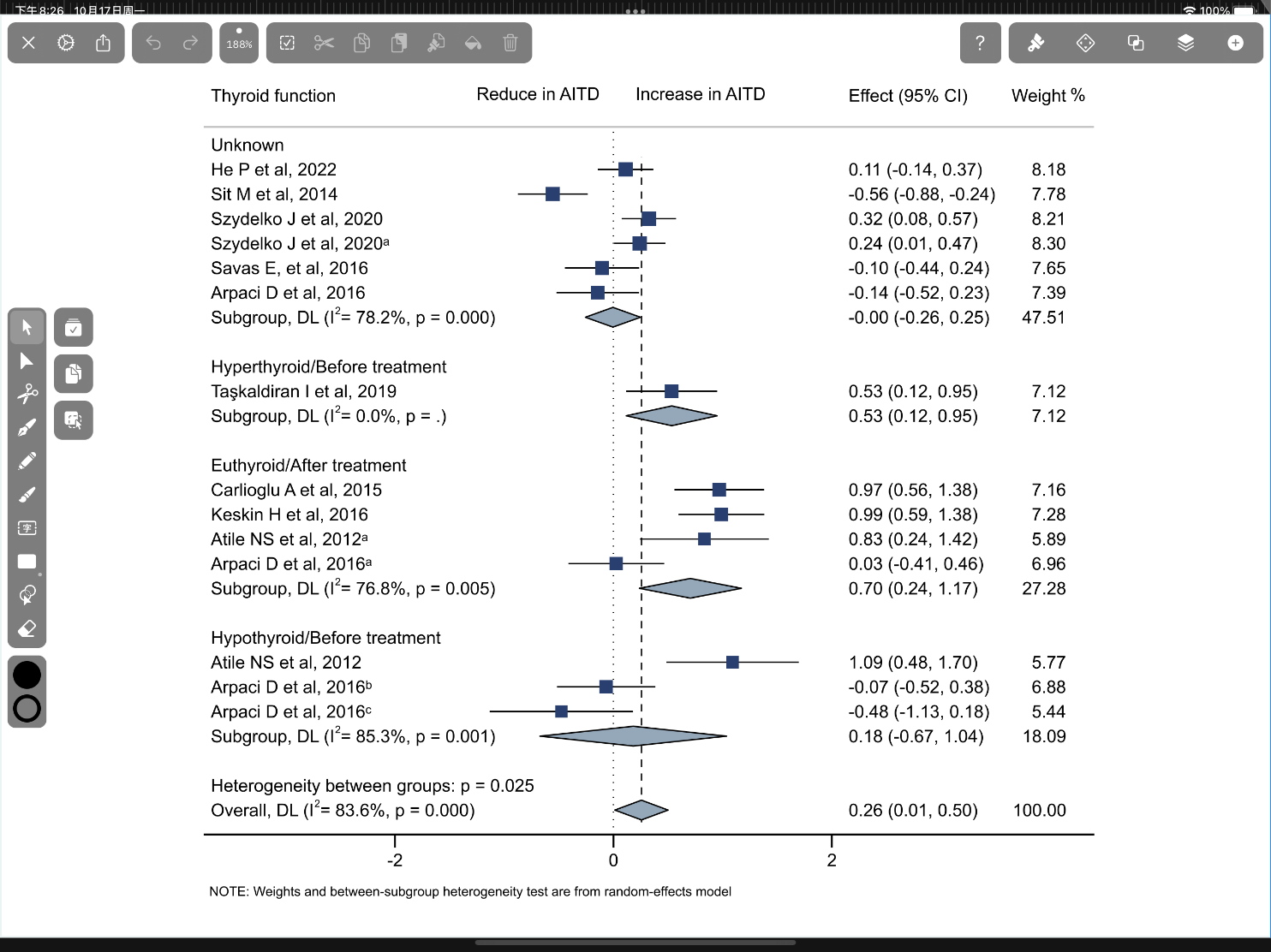


C study type

**
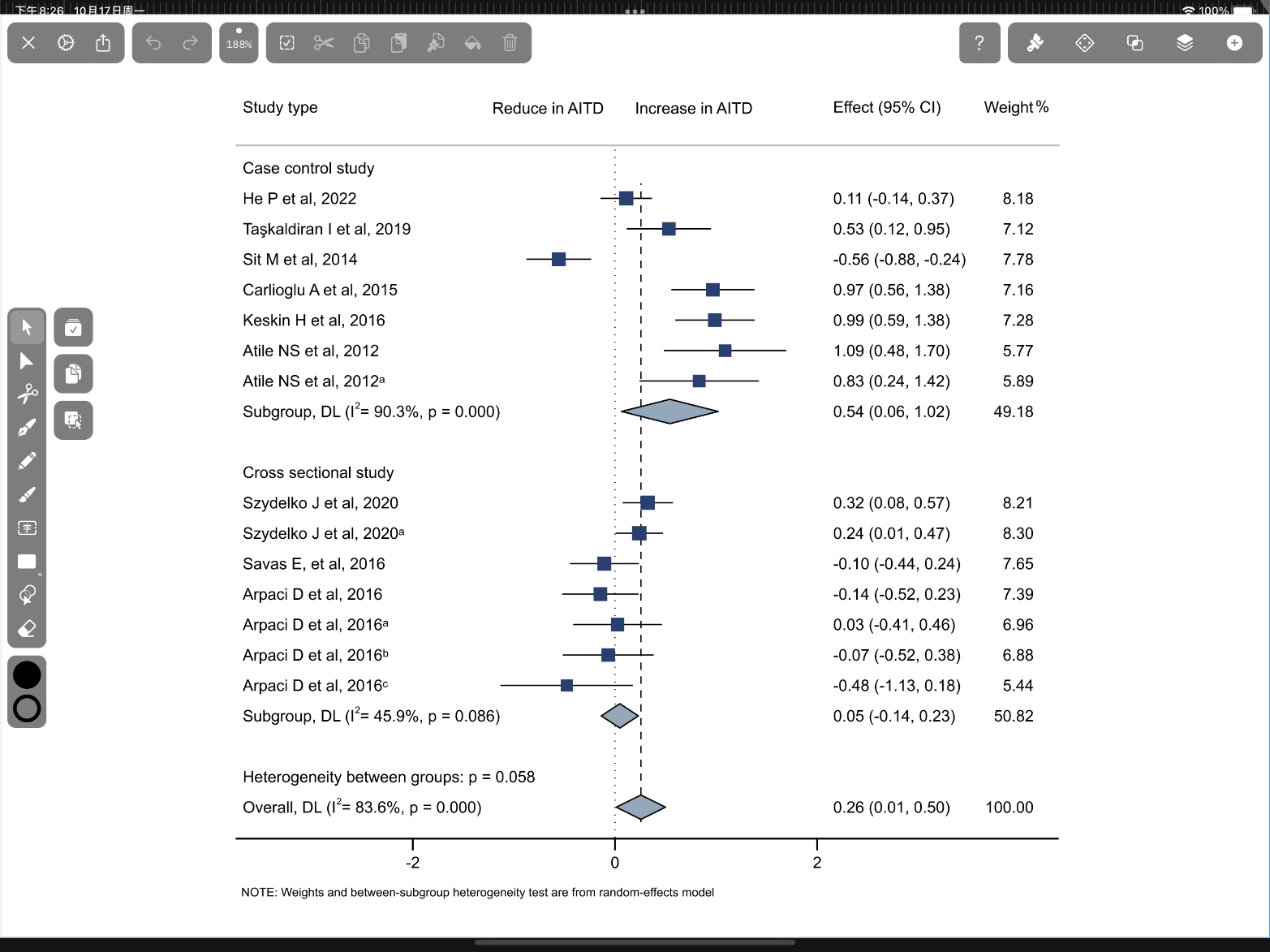
**

Abbreviations: GD, Graves’ disease; HT, Hashimoto’s thyroiditis; AITD, autoimmune thyroid disease; MPV, mean platelet volume; 95% CI: 95% confidence interval

^a^: the second available data from the same study

^b^: the third available data from the same study

^c^: the fourth available data from the same study

**Supplementary Figure S3.** Funnel Plot

A. PLT


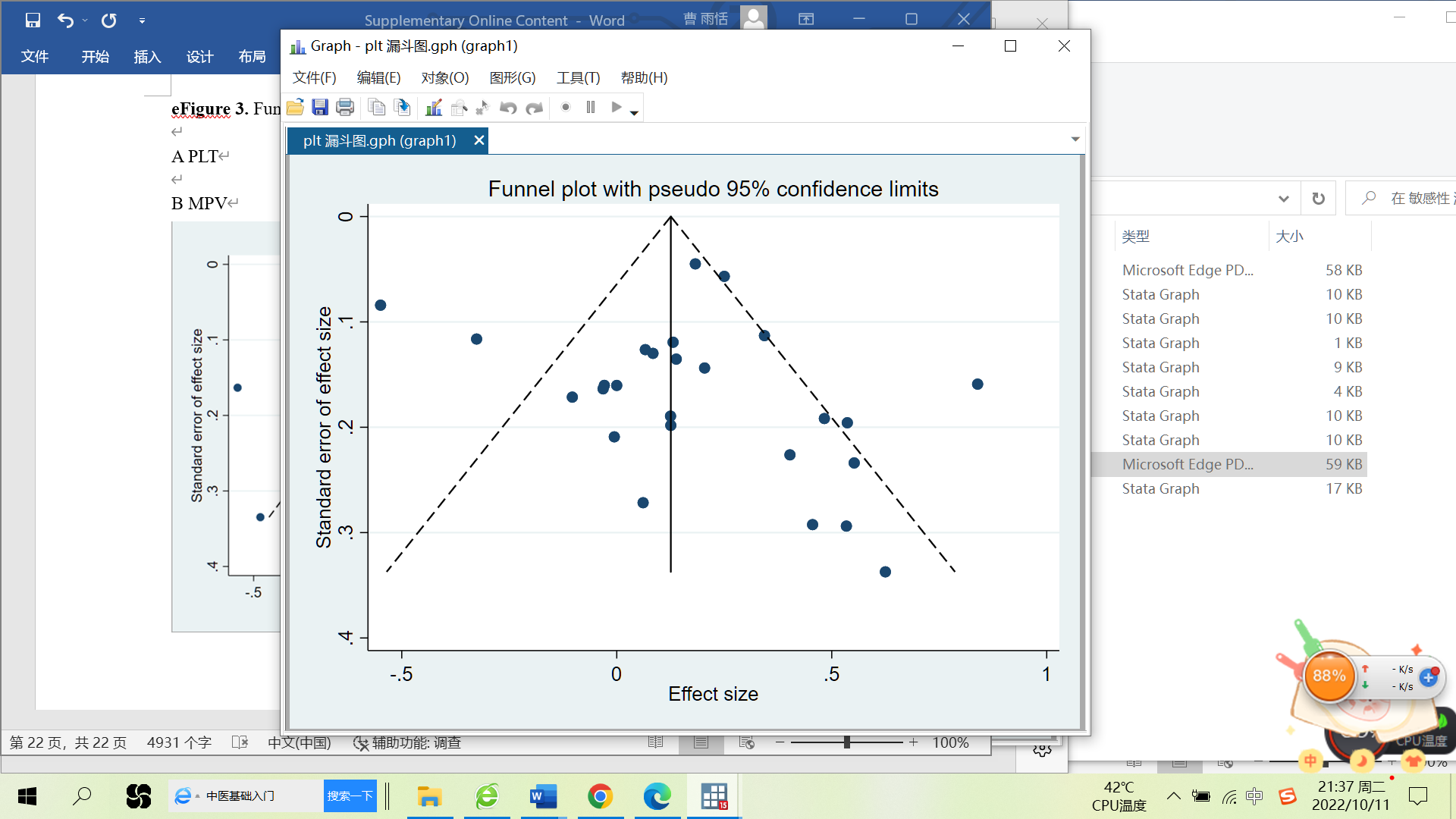


B. MPV


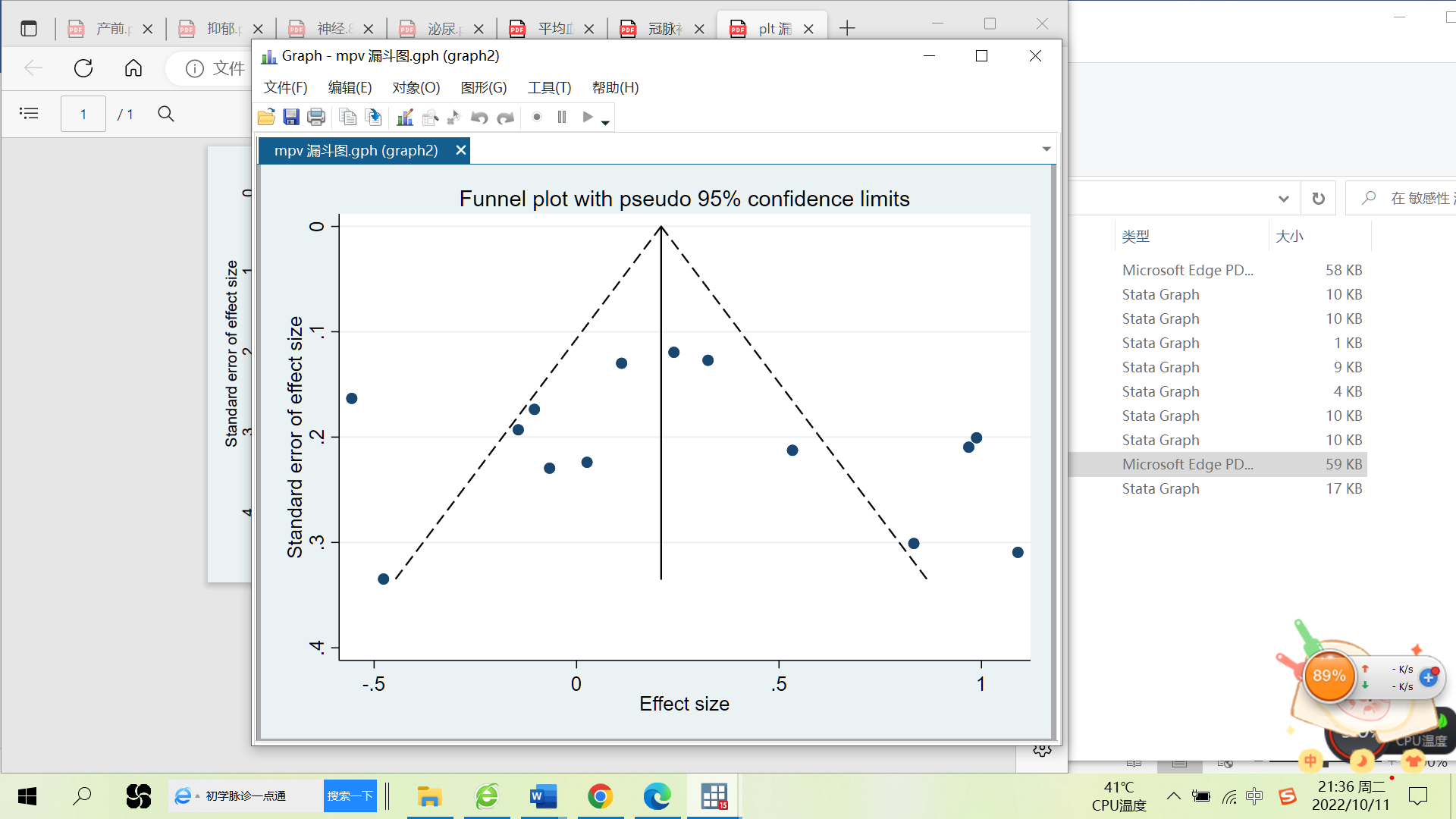


Abbreviations: PLT, platelet count; MPV, mean platelet volume

**Supplementary Figure S4.** Egger’s Test

A PLT


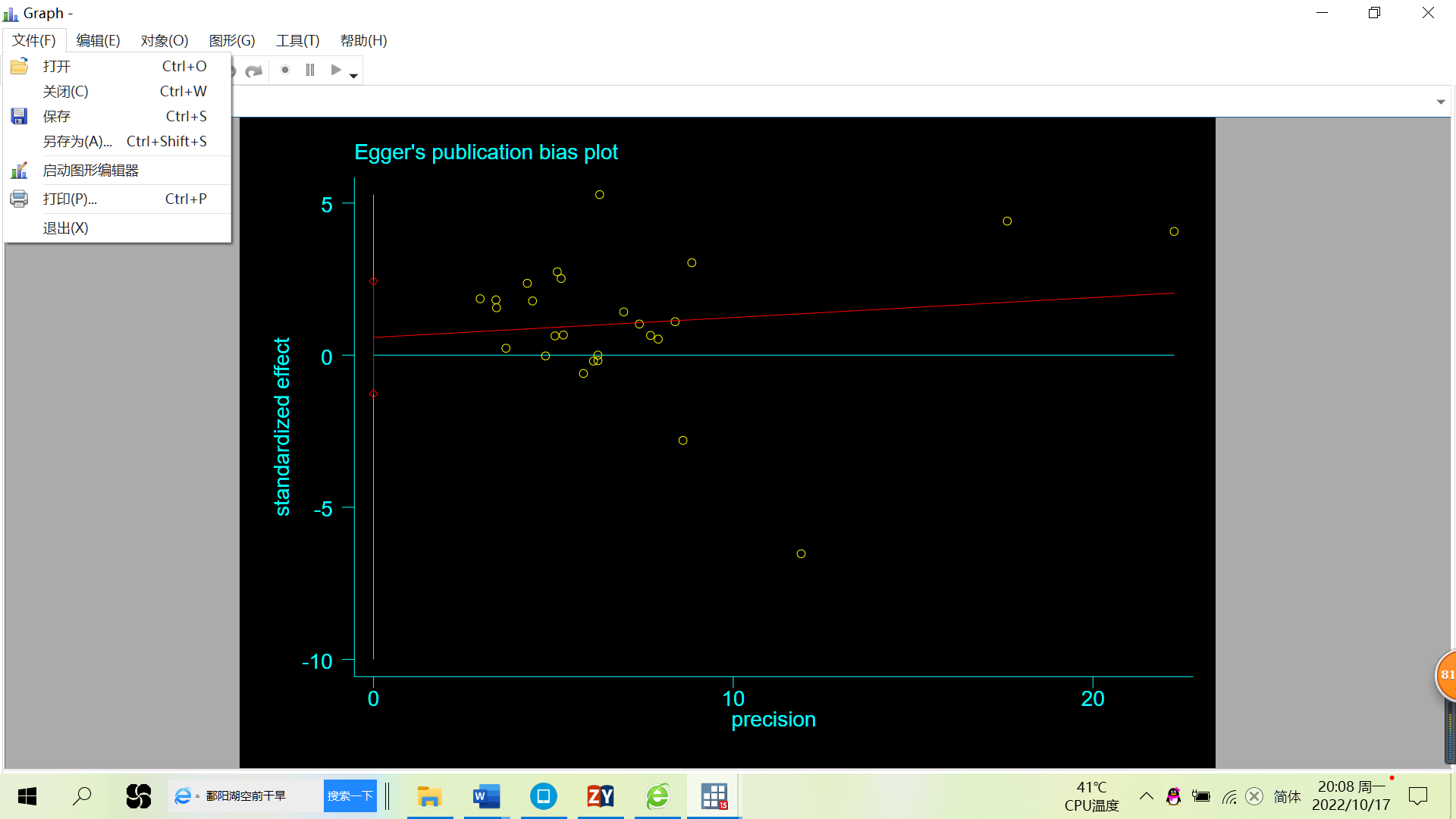


B MPV


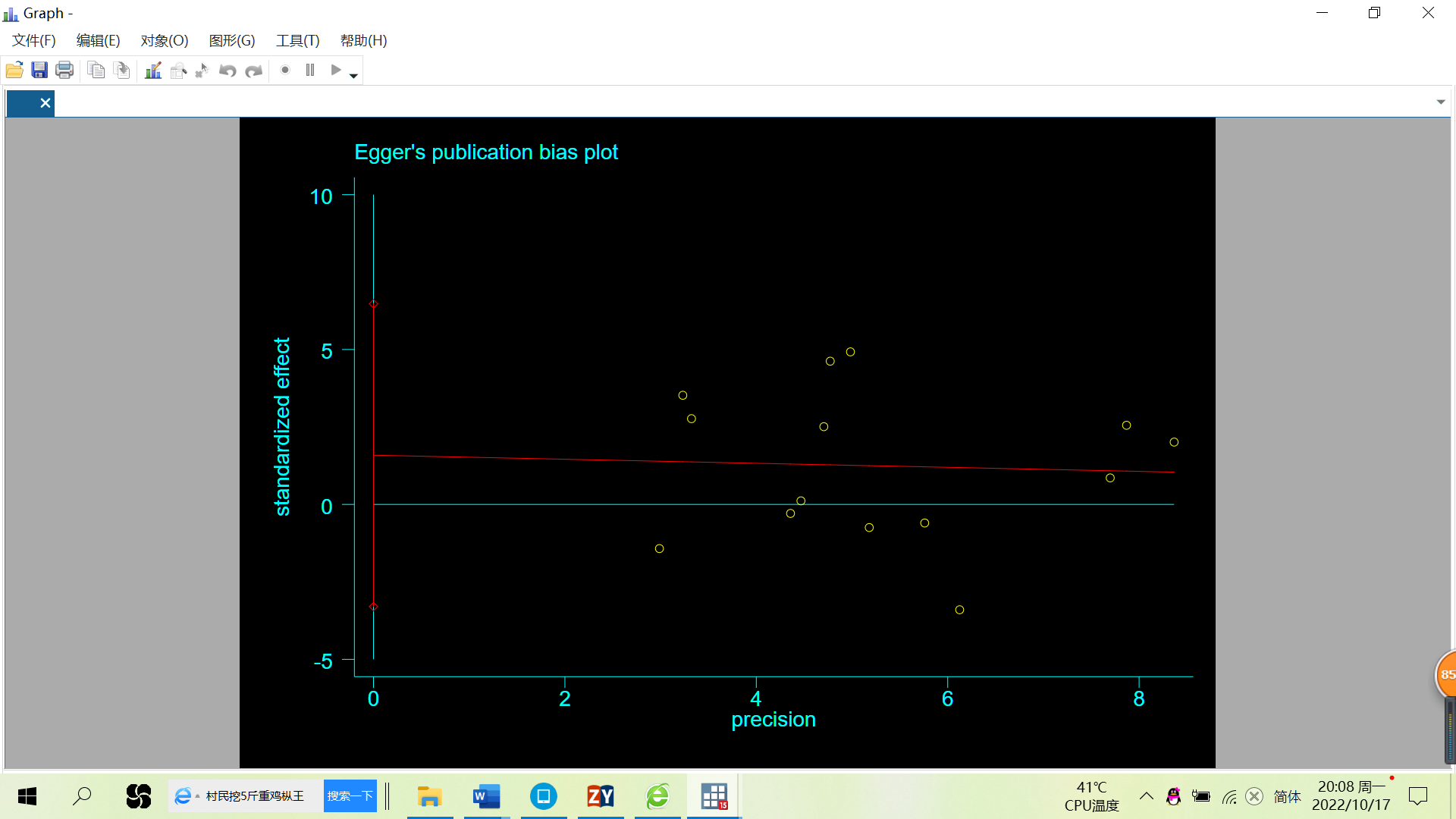


Abbreviations: PLT, platelet count; MPV, mean platelet volume
